# Bioengineered liver crosslinked with nano-graphene oxide enables efficient liver regeneration via MMP suppression and immunomodulation

**DOI:** 10.1038/s41467-023-35941-2

**Published:** 2023-02-13

**Authors:** Da-Hyun Kim, Min-Ji Kim, Seon-Yeong Kwak, Jaemin Jeong, Dongho Choi, Soon Won Choi, Jaechul Ryu, Kyung-Sun Kang

**Affiliations:** 1 Adult Stem Cell Research Center and Research Institute for Veterinary Medicine, College of Veterinary Medicine, Seoul National University, Seoul, 08826 Republic of Korea; 2 Department of Agriculture, Forestry and Life Science, College of Agriculture and Life Science, Seoul National University, Seoul, 08826 Republic of Korea; 3 Bio-MAX Institute, Seoul National University, Seoul, 08826 Republic of Korea; 4 Department of Surgery, Hanyang University College of Medicine, Seoul, 04763 Republic of Korea; 5 Institute of Bio & Nano Convergence, Biogo Co., LTD, Seoul, 08826 Republic of Korea

**Keywords:** Tissue engineering, Biomedical materials, Biomedical materials, Biomedical engineering, Regenerative medicine

## Abstract

Decellularized extracellular matrix scaffold, widely utilized for organ engineering, often undergoes matrix decomposition after transplantation and produces byproducts that cause inflammation, leading to clinical failure. Here we propose a strategy using nano-graphene oxide to modify the biophysical properties of decellularized liver scaffolds. Notably, we demonstrate that scaffolds crosslinked with nano-graphene oxide show high resistance to enzymatic degradation via direct inhibition of matrix metalloproteinase activity and increased mechanical rigidity. We find that M2-like macrophage polarization is promoted within the crosslinked scaffolds, which reduces graft-elicited inflammation. Moreover, we show that low activities of matrix metalloproteinases, attributed to both nano-graphene oxide and tissue inhibitors of metalloproteinases expressed by M2c, can protect the crosslinked scaffolds against in vivo degradation. Lastly, we demonstrate that bioengineered livers fabricated with the crosslinked scaffolds remain functional, thereby effectively regenerating damaged livers after transplantation into liver failure mouse models. Overall, nano-graphene oxide crosslinking prolongs allograft survival and ultimately improves therapeutic effects of bioengineered livers, which offer an alternative for donor organs.

## Abstract (web summary)

Bioengineered livers using decellularized scaffolds have been considered as an alternative to donor organs. Here, the authors modulate biophysical properties of decellularized scaffolds by crosslinking with nano-graphene oxide, thereby greatly enhancing therapeutic efficacy of bioengineered livers.

## Introduction

Liver transplantation is the ultimate life-saving treatment for end‐stage liver diseases to date, which has been hampered by a critical shortage of organs^1^. As an alternative to donor organs, many bioinspired approaches have been widely explored for liver tissue engineering, such as cell encapsulation, 3D printing and hepatic organoids, in combination with natural or synthetic materials (e.g., hydrogel, alginate, gelatin methacryloyl (GelMA), and polycaprolactone)^2,3^. However, such approaches have limitations in closely restoring the highly sophisticated microstructures and biochemical properties of the liver. In this light, a decellularized extracellular matrix (dECM) scaffold in which all cells are removed from the organ but the organ-specific architectures and biochemical niches remain has been a promising substrate for liver tissue engineering^4,5^. Using allograft-derived scaffolds would be more clinically relevant, but only a very limited number of human organs can be obtained; hence, organs from other species have also been utilized^6,7^. Ultimately, bioengineered livers produced with dECM scaffolds and liver-composing cells have been extensively developed to replace or regenerate injured liver tissues^8,9^.

Despite these attempts, the clinical applications of bioengineered tissues to human patients have been hampered by several obstacles. Above all, the dECM liver scaffolds are vulnerable to in vivo degradation owing to their mechanical fragility, which leads to the loss of the physical and biochemical microenvironment of encapsulated cells. In addition, since genetic and phenomic features of matrisomes can be variant across species^10^, dECM scaffolds derived from other species also have potential for immunogenicity due to residues of the original species^11^. Thus, upon transplantation of xenograft-derived scaffolds, innate host immune systems are activated to eliminate foreign materials^12^. For example, neutrophils are the first responders taking part in phagocytosis and degranulation after infiltration into implanted biomaterials^13,14^. Following that, circulating monocytes are also recruited into the tissues and differentiated into macrophages, which dictate both inflammatory responses and tissue remodeling in the implants^12,15^. These infiltrating host cells, such as neutrophils, macrophages, lymphocytes, and stromal cells, continuously secrete matrix metalloproteinases (MMPs), which catalyze the ECM components of the scaffolds^11,16^. As a consequence of matrix degradation, bioactive ECM byproducts, termed “matricryptins” or “matrikines”, are produced and propagate further inflammatory cascades due to their chemotactic properties^17^. Eventually, a series of immune responses, which are elicited by both matricryptins arising from ECM degradation and the intact forms of the ECM components, may lower therapeutic effects and result in clinical failure.

In light of these challenges, there has been a great need for the development of potent and biocompatible crosslinking agents to protect bioscaffolds from both mechanical deformation and immune responses following implantation. To reinforce the mechanical characteristics of scaffolds, glutaraldehyde (GA) has been developed as a potent crosslinking agent, but its clinical applicability has been limited because of its complications, including cytotoxicity and calcification^18^. To date, genipin^19^ and carbodiimide^20^ have also been utilized as biocompatible crosslinking agents, but achieving high effectiveness comparable to the effectiveness of GA remains unresolved. In addition, recent strategies for reducing the immunogenicity of the dECM scaffold itself have been studied, such as genetic manipulation of organs^21^, or regulation of the inflammatory milieu of bioengineered constructs^17^.

Graphene, a sheet of carbon atoms packed in a honeycomb lattice, exhibits different features depending on their size and functional moieties. Graphene oxide (GO) is a chemically modified graphene with the oxygen-containing functional groups (carboxyl, hydroxyl and epoxy groups). Nano-graphene oxide (NGO) with lateral dimensions below 100 nm and a few layers, takes advantage of quantum confinement and edge effects over GO^22^. Owing to their biocompatibility and immunomodulatory effects, significant progress has been made in adopting NGOs for biomedical purposes^23,24^. However, despite several attempts to use GO for reinforcing biopolymers^25,26^, the in vivo effects of scaffolds crosslinked with NGOs and moreover, bioengineered organs with NGO-crosslinked scaffolds have not been elaborated yet.

In this study, the primary objective was to produce a bioengineered liver using dECM scaffolds with enhanced physical and biochemical properties. By utilizing the NGOs, we achieved a highly resistant dECM scaffold against mechanical deformation and MMP-mediated enzymatic degradation. We also elucidated the molecular interactions between NGOs and MMPs. Next, the crosslinked scaffolds were transplanted into the mice for up to 60 days and evaluated the in vivo durability and immune responses provoked by the implants. Ultimately, mouse bioengineered livers (MBLs) using the NGO-crosslinked scaffolds showed superior regenerative potential of the MBLs when transplanted into an acute or chronic liver failure model. Altogether, we proposed that the NGOs play noteworthy roles in promoting the structural stability of scaffolds, alleviating pro-inflammatory responses by regulating macrophage polarity toward M2c. Furthermore, NGO crosslinking enabled generation of functional MBLs which promoted restoration of impaired liver functions in vivo.

## Results

### Crosslinking of dECM liver scaffolds with NGOs

**Fig. 1:**
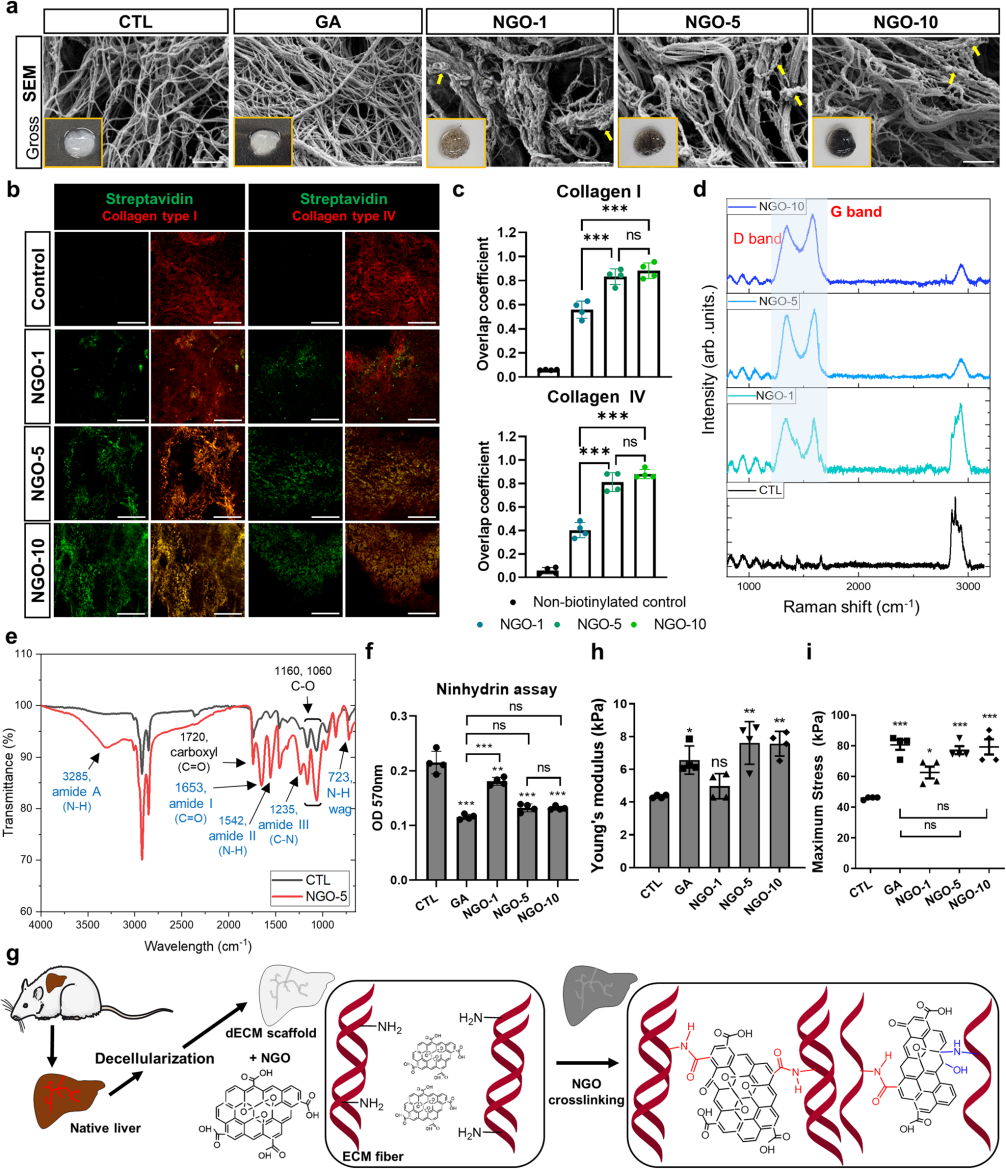
NGO crosslinking contributes to improvement of physical properties of decellularized scaffolds via amide bond formation. **a** Scanning electron microscopy (SEM) analysis and macroscopic images of the scaffolds crosslinked with PBS (CTL), 0.625% glutaraldehyde (GA), and nano-graphene oxide (NGO): 1 μg/mL (NGO-1), 5 μg/mL (NGO-5) and 10 μg/mL (NGO-10). Note the NGOs attached to the surface of extracellular matrix (ECM) fibrils (yellow arrow). Scale bar, 1 µm. **b** Representative confocal images of the NGO-crosslinked scaffolds probed with streptavidin (green), Collagen type I and type IV, respectively (red). The scaffolds crosslinked with 10 μg/mL of non-biotinylated NGOs were used as negative control. Scale bar, 200 µm. **c** Overlap coefficient measuring the degree of colocalization between Streptavidin^+^ NGOs and Collagen I^+^ or Collagen IV^+^ ECM fibers (*n* = 4). **d** Raman spectroscopy analysis of CTL scaffold and NGO-crosslinked scaffolds. **e** Fourier transform infrared spectroscopy (FTIR) transmittance spectra of CTL scaffold and the scaffold crosslinked with NGO-5. **f** Ninhydrin assay for quantifying the free amine groups of scaffolds in each group (*n* = 4). **g** Schematic illustration of chemical crosslinking decellularized ECM scaffolds with NGOs. **h** Young’s modulus of each scaffold in 5% strain (*n* = 4). **i** Maximum amount of stress that each scaffold could withstand (*n* = 4). Quantitative data were presented as a mean ± SD. Statistical differences between the groups were determined by ordinary one-way ANOVA with post-hoc Tukey test (*p < 0.05, **p < 0.01, ***p < 0.001 versus CTL, ns; not statistically significant). Source data are provided as a Source Data file.

First, the chemical properties of NGOs synthesized for the purpose of crosslinking were characterized. The spherical morphology of 5-layered NGO sheets was visualized by high resolution transmission electron microscopy (HRTEM) (Supplementary Fig. 1a). Although scanning electron microscopy (SEM) analysis revealed that the dimension of the particles ranged from 13 to 43 nm (Supplementary Fig. 1b), the representative size of the NGOs was determined to be 28.2 nm (Supplementary Fig. 1c). The thickness of NGOs ranged from 3 to 8 nm (Supplementary Fig. 1d), and the distance between graphene layers was estimated to be 8.57Å **(**Supplementary Fig. 1e). The peaks in Raman spectrum of the NGOs appeared at approximately 1350 cm^−1^ (D band) and 1580 cm^−1^ (G band), which are characteristic peaks of the graphene (Supplementary Fig. 1f). Using surface analysis, C–C (51.4%), C–O (38.76%) and C=O (9.85%) bonds, specifically 65.01 wt% of C and 33.92 wt% of O, was identified in the NGOs (Supplementary Fig. 1g). Fourier transform infrared spectroscopy (FTIR) spectrum of the NGOs showed the peaks all corresponding to carbohydrate moieties (Supplementary Fig. 1h). A zeta potential value of −38.6 ± 6.86 mV indicated the moderate dispersion stability of the NGOs (Supplementary Fig. 1i). Prior to crosslinking, we conducted histological examinations to verify the preservation of ECM structures without preexisting host cells in the dECM scaffolds (Supplementary Fig. 2a–d). Compared to native livers, DNA contents were significantly reduced (Supplementary Fig. 2e), while the ECM contents were preserved in the dECM scaffolds (Supplementary Fig. 2f). According to the liquid chromatography/ mass spectrometry (LC/MS) analysis of each liver tissue, only 0.39% of proteins were matrisome proteins in the native liver, while 47.35% of matrisome proteins were presented in the dECM liver scaffold. Among matrisome proteins, 71.56% of core matrisome and 18.37% of matrisome-associated proteins were found in the dECM liver. In contrast, matrisome-associated proteins accounted for 84.39%, constituting most of the matrisome proteins in the native livers. In both groups, core matrisome consisted mostly of ECM glycoproteins and collagens, and the ECM-affiliated proteins accounted for the largest amount among the matrisome-associated proteins (Supplementary Fig. 2g, h, Supplementary Data 1). The heatmap demonstrating the relative expression of top 30 core matrisome proteins identified that biglycan, fibronectin and collagen were the main ECM components in the dECM liver scaffolds (Supplementary Fig. 2i, Supplementary Data 1). Next, dECM scaffolds were perfused with PBS (CTL, negative control), 0.625% GA (positive control) and different concentrations of NGOs for crosslinking. Due to the intricate microstructures of the liver, perfusion crosslinking via both portal vein and bile duct was beneficial for distributing NGOs evenly throughout dECM scaffolds, according to the previous report^27^. After crosslinking, SEM analysis showed NGOs bound to the ECM fibrils (Fig. 1a). Compared to the perfusion solution before crosslinking, the D and G bands in the Raman spectra were not found in the perfusion solutions after crosslinking, indicating that all graphene particles remained within the scaffolds and no longer flowed out of the scaffolds (Supplementary Fig. 3a). As demonstrated by the overlap coefficient between NGOs and collagen fibers, 83.23 ± 0.07% of collagen I and 81.28 ± 0.08% of collagen IV were respectively co-localized with NGOs in the NGO-5 group, which showed no significant difference with those in the NGO-10 group (Fig. 1b, c).

### Chemical and physical properties of the NGO-crosslinked scaffolds

We next analyzed the chemical mechanisms of NGO crosslinking of the dECM scaffolds. Regardless of NGO concentration, Raman spectra of the NGO-crosslinked scaffolds showed the D and G bands, suggesting the incorporation of NGOs in the scaffolds (Fig. 1d). These bands were not detected in the GA-crosslinked scaffolds (Supplementary Fig. 3b). The oxygen-containing functional groups of the NGOs, such as carboxylic acid, epoxide and carbonyl groups, can simultaneously react with the primary amine groups of the proteins^28^. ATR (attenuated total reflection)-FTIR analysis allowed us to quantitatively compare the chemical bonding changes in the crosslinked scaffolds. The new peaks at 3285, 1653, 1542 and 1235 cm^−1^ were identified in the ATR-FTIR spectra of the NGO-crosslinked scaffolds, indicating the formation of amide A (N-H stretching), amide I (C=O stretching), amide II (N-H bending) and amide III (C-N stretching) bonds^29^ (Fig. 1e). These results indicated that carboxylic acid groups of the NGOs were reacted with primary amine groups of the ECM proteins, resulting in the formation of amide bonds. Second, the ring-opening reaction between epoxide of NGOs and primary amines of the scaffolds also occurred as evidenced by the N-H wagging peak of the secondary amine bond at 723 cm^−1^ in the ATR-FTIR spectra (Fig. 1e). However, the intensity of the N-H wagging peak was not as high as that of the amide peaks, suggesting the lower reactivity of epoxide compared to that of carboxylic acid in the NGOs^28^. Furthermore, the scaffolds crosslinked with different concentrations of NGOs showed exactly the same patterns of chemical bonds, but the intensity of those amide peaks was markedly increased in the NGO-5 and NGO-10 groups compared to the NGO-1 group (Supplementary Fig. 3c). Indeed, ninhydrin assay measuring the amount of free primary amine groups available for reactions showed that the crosslinking degree of the NGO-5 and NGO-10 groups was significantly increased, which was comparable to that of GA crosslinking (Fig. 1f). Taken together, the primary amine groups in the dECM reacted mainly with carboxyl groups and partially with epoxy rings of the NGOs, and formed amide bonds during the NGO crosslinking of dECM scaffolds (Fig. 1g). Next, to verify our hypothesis that NGO crosslinking could enhance the physical properties of the dECM scaffolds, Young’s modulus of each scaffold was measured. Although a low concentration of NGO (NGO-1) was ineffective, the elastic modulus of the NGO-5 and NGO-10 groups was comparable to that of the GA group and much higher than that of the CTL group (Fig. 1h, Supplementary Fig. 3d). Moreover, the ultimate tensile strength, demonstrated as the maximum load that scaffolds could endure, was higher in the NGO-5 and NGO-10 scaffolds than in the CTL scaffolds (Fig. 1i). Collectively, we achieved high resistance of the dECM scaffolds to mechanical transformation as a consequence of NGO crosslinking, which was comparable to GA crosslinking.

### Protective effects of NGO-crosslinked scaffolds from in vitro degradation

**Fig. 2:**
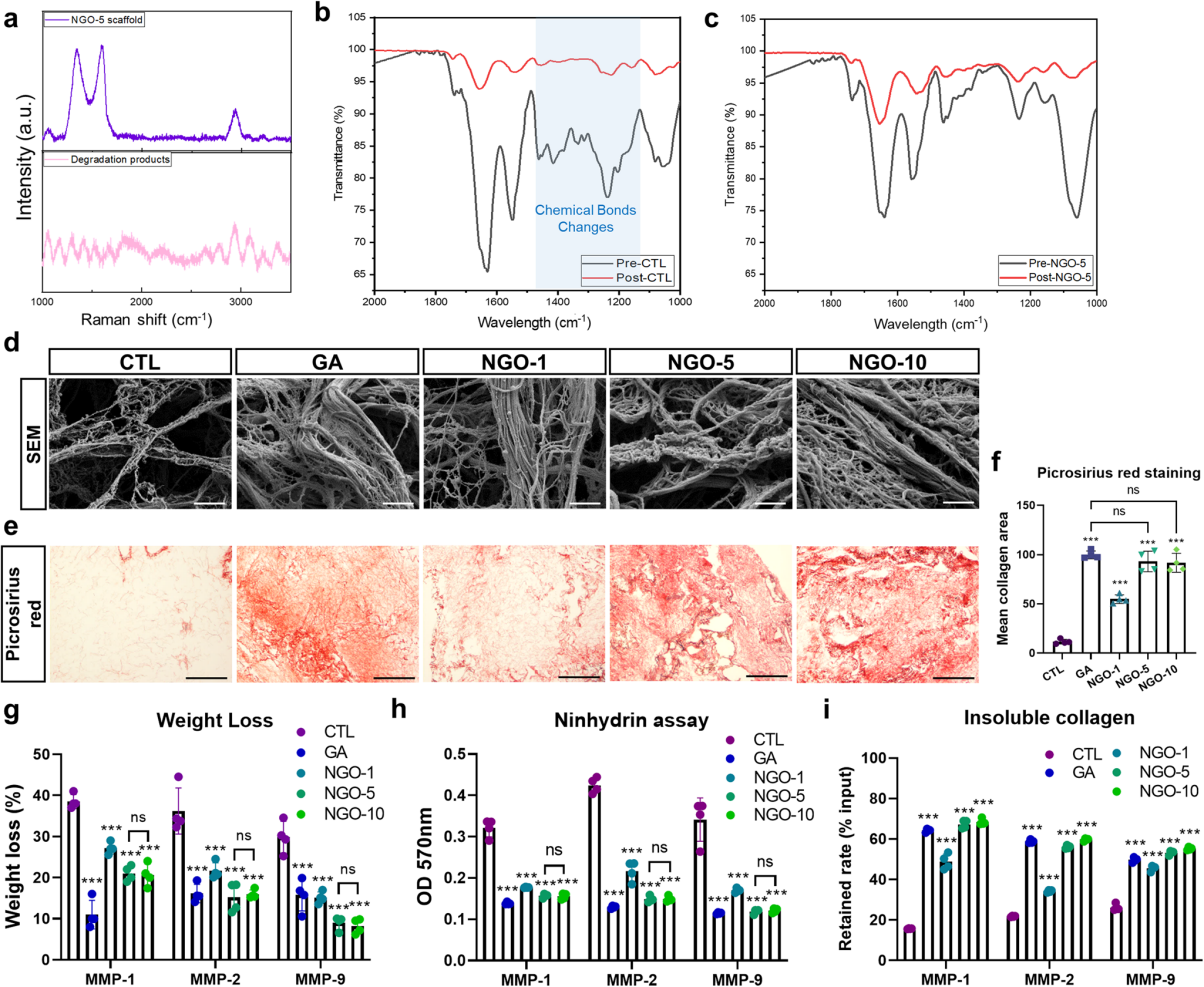
In vitro resistance of NGO-crosslinked scaffolds to enzymatic degradation. **a** Raman spectra of scaffold crosslinked with 5 μg/mL nano-graphene oxide (NGO-5) after MMP-1 treatment (top) and degradation products of MMP-treated scaffold (bottom). **b,c** Fourier transform infrared spectroscopy (FTIR) spectra of non-crosslinked scaffolds (CTL) and NGO-5 scaffolds were measured before (black) and after (red) MMP-1 treatment. **d** Scanning electron microscopy (SEM) images of the crosslinked scaffolds after MMP-1 treatment for 2 h. Scale bar, 1 µm. **e** Picrosirius red staining of the scaffolds degraded by MMP-1. Scale bar, 200 µm. **f** Area quantification of collagen fibers stained by picrosirius red (*n* = 4). **g** Weight loss measurements of each scaffold after treatment of MMP-1, MMP-2 and MMP-9 (*n* = 4). **h** Ninhydrin assay quantifying the crosslinking degree of the scaffolds after MMP treatment (*n* = 4). **i** Quantification of the amount of retained insoluble collagen of the scaffolds exposed to MMPs normalized to that of intact scaffolds (*n* = 4). Quantitative data were presented as a mean ± SD. Statistical differences between the groups were determined by ordinary one-way ANOVA with post-hoc Tukey test (***p < 0.001 versus CTL, ns; not statistically significant). Source data are provided as a Source Data file.

After dECM scaffold transplantation, various types of cells, including macrophages, neutrophils and stromal cells, are recruited into the scaffolds. Such cells regulate the matrix degradation and turnover by secreting proteases like MMPs^14,16^. Hence, it is critical to assess whether NGO-crosslinked scaffolds can protect ECM structures from enzymatic degradation in addition to physical strain. Considering that collagen and fibronectin mainly comprised the core matrisome of dECM scaffolds (Supplementary Fig. 2i), we attempted to recapitulate the in vitro enzyme-mediated degradation of the dECM scaffolds by exploiting MMP-1, MMP-2 and MMP-9, which could cleave collagen and fibronectin. The Raman spectrum of the NGO-5 scaffold still showed the D and G bands after MMP-1 exposure, indicating that the NGOs remained intact within the scaffold after ECM decomposition (Fig. 2a). In contrast, no characteristic peaks of the NGOs were detected in the degradation product of NGO-5 and NGO-10, implying that incorporated NGOs were unseparated from the scaffolds during ECM degradation due to stable amide bonds (Supplementary Fig. 3e). Furthermore, the patterns of FTIR spectra in the NGO-5 group were less affected by MMP-1 exposure than those in the CTL and NGO-1 groups showing numerous broken chemical bonds (Fig. 2b, c, Supplementary Fig. 3f). Thus, dECM scaffolds crosslinked with at least 5 μg/mL NGO were found to be less prone to degradation. Both SEM analysis and picrosirius red staining results also revealed that the microstructures of NGO- or GA-crosslinked scaffolds after MMP-1 treatment were relatively preserved in contrast with those of the CTL scaffolds showing the strong dissociation of ECM fibers (Fig. 2d–f). Next, quantitative analyses of the protective effects of the NGO crosslinking against various MMPs (MMP-1, 2 and 9) were conducted. The weight loss of the CTL scaffolds after exposure to MMPs was significantly larger than that of the crosslinked scaffolds (Fig. 2g). Ninhydrin assay also showed that the crosslinking degree of the NGO-5 and NGO-10 scaffolds after MMP treatment was maintained intact, which was as high as that of the GA-crosslinked scaffolds (Fig. 2h). After MMP treatment, the amount of remaining insoluble collagen, normalized to dry weight of each scaffold, in the NGO-crosslinked scaffolds was comparable to that in the GA-crosslinked scaffold (Fig. 2i, Supplementary Fig. 3g). Taken together, NGO crosslinking contributed to acquiring high resistance of dECM scaffolds against MMP-mediated degradation and mitigating the loss of ECM components.

### Inhibitory effects of NGOs on MMP activities via direct interaction

**Fig. 3:**
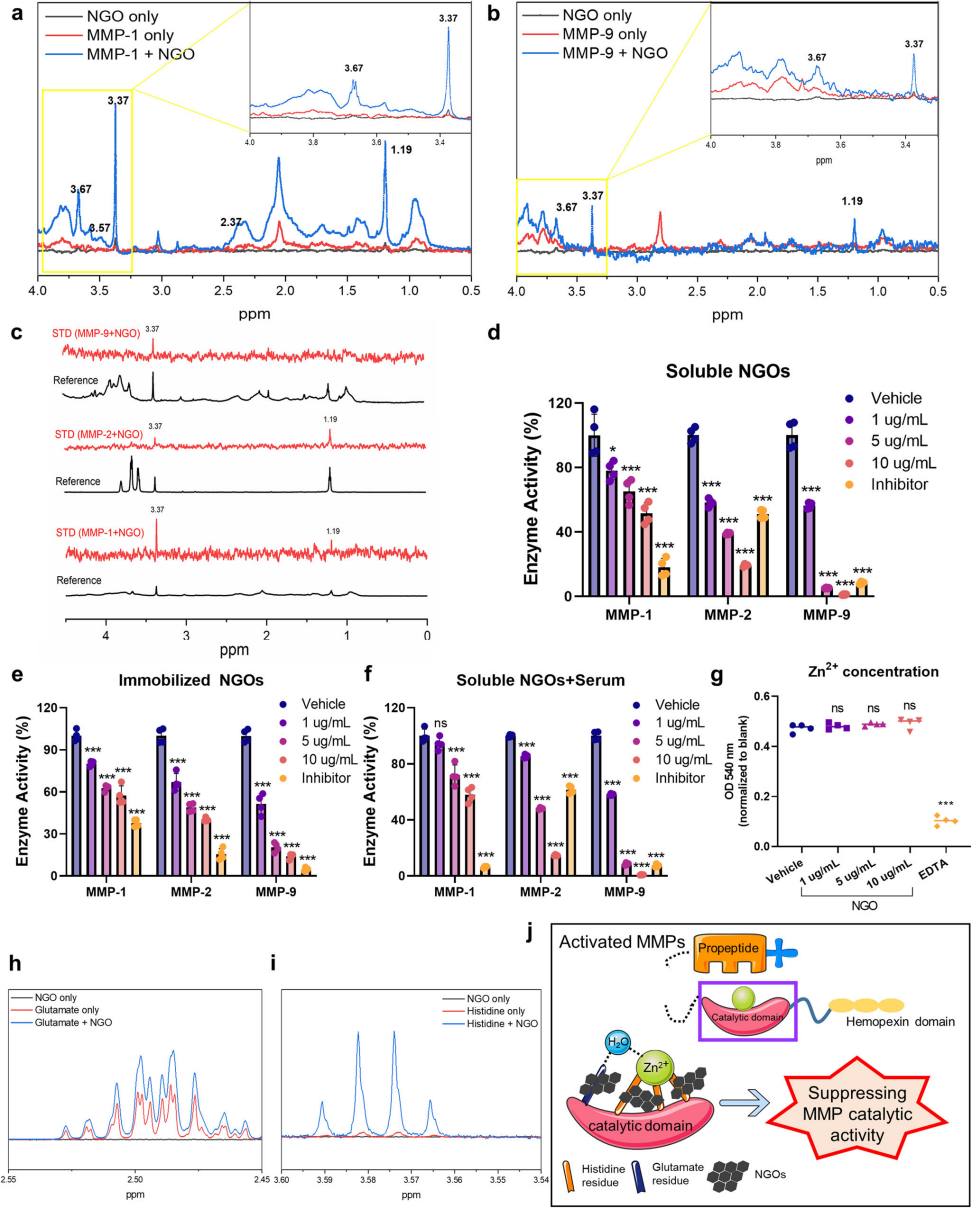
NGOs suppress MMP activities via direct binding to catalytic domain of MMPs. **a**, **b** ^1^H nuclear magnetic resonance (NMR) spectra. Nano-graphene oxide (NGO) only, MMPs only (MMP-1, MMP-9) and MMPs incubated with NGOs (MMP + NGO). **c** Saturation transfer difference (STD)-NMR spectra of MMP-1, MMP-2 and MMP-9 in the presence of NGOs (red) and the respective reference spectra (black). **d**–**f** Catalytic activities of MMP-1, MMP-2 and MMP-9 incubated with 1 μg/mL, 5 μg/mL and 10 μg/mL of NGOs dispersed in distilled water **(d)**, immobilized within the scaffolds **(e)**, or incubated with serum proteins **(f)**. Vehicle (distilled water) and inhibitors (1,10-Phenanthroline for MMP-1 inhibition and doxycycline for MMP-2 and MMP-9 inhibition) were used (*n* = 4). **g** Zinc chelating activity of NGOs at different doses. 10 µM of EDTA was used for a positive control (*n* = 4). **h**, **i** ^1^H NMR spectra of NGO only, glutamate/ histidine only, and glutamate/ histidine incubated with NGOs (glutamate/ histidine + NGO). **j** Graphical scheme of interactions between MMPs and NGOs. Quantitative data were presented as a mean ± SD. Statistical differences between the groups were determined by ordinary one-way ANOVA with post-hoc Tukey test (*p < 0.05, **p < 0.01, ***p < 0.001, ns; not statistically significant versus Vehicle). Source data are provided as a Source Data file.

We further attempted to unravel the fundamental mechanism by which the NGOs could protect against the breakdown of the dECM scaffolds. The interactions between the NGO particles and enzymatically-activated MMPs, which were prepared by cleaving pro-peptides and exposing catalytic domains of the zymogens^30^, were examined using nuclear magnetic resonance (NMR). As a result, we identified chemical shifts in the ^1^H NMR spectra of MMP-1 and MMP-9 after incubation with NGO, reflecting the presence of interactions between the NGOs and MMPs (Fig. 3a, b). In addition, the peaks in the saturation transfer difference (STD)-NMR spectra of the MMPs (MMP-1, MMP-2 and MMP-9) incubated with NGOs provided strong evidence that the NGOs directly interacted with activated MMPs (Fig. 3c). Notably, these interactions occurred in the same regions (1.19 and 3.37 ppm) regardless of the MMP subtype, suggesting that NGOs likely bind to the conserved region of MMPs. Furthermore, the NGO particles that were not only dispersed in solution but also immobilized within the dECM scaffolds significantly reduced the catalytic activities of MMPs in a dose-dependent manner (Fig. 3d, e). Considering that in vivo reactivity of NGOs could be influenced by serum proteins adsorbed on the graphene surface^31^, we also confirmed that NGOs inhibited MMP catalysis even in the presence of serum proteins (Fig. 3f). Since graphene has been known to affect catalytic performances via enzyme immobilization by modulating the intrinsic structural properties of the enzymes^32^, we identified that direct binding of NGOs to the catalytic domain and eventually led to suppression of the catalytic activities of MMPs. The MMP catalytic domains harbor catalytic Zn^2+^ ions and their binding motif (HExxHxxGxxH) are both responsible for structural coordination and highly conserved among different MMP subtypes^30^. To further identify the binding sites of NGOs within the catalytic domain, we first proved that NGOs could not directly chelate zinc (Fig. 3g). Next, we examined whether NGOs influenced the conserved amino acids, histidine (H) and glutamate (G), in the zinc-binding motif. Several newly appeared peaks in the ^1^H NMR spectra of NGOs incubated with each amino acid indicated that NGOs interacted with both glutamate and histidine (Fig. 3h, i). In sum, NGO inhibited the enzymatic activities of MMPs via direct binding to conserved motifs in the catalytic domains (Fig. 3j). Such approaches not only provided relevant information on the enzymatic susceptibility of NGO-crosslinked scaffolds, but also unraveled the fundamental mechanism of how NGOs can affect other MMP-mediated pathophysiological events.

### NGO crosslinking alleviates scaffold degradation and inflammatory responses after transplantation

**Fig. 4:**
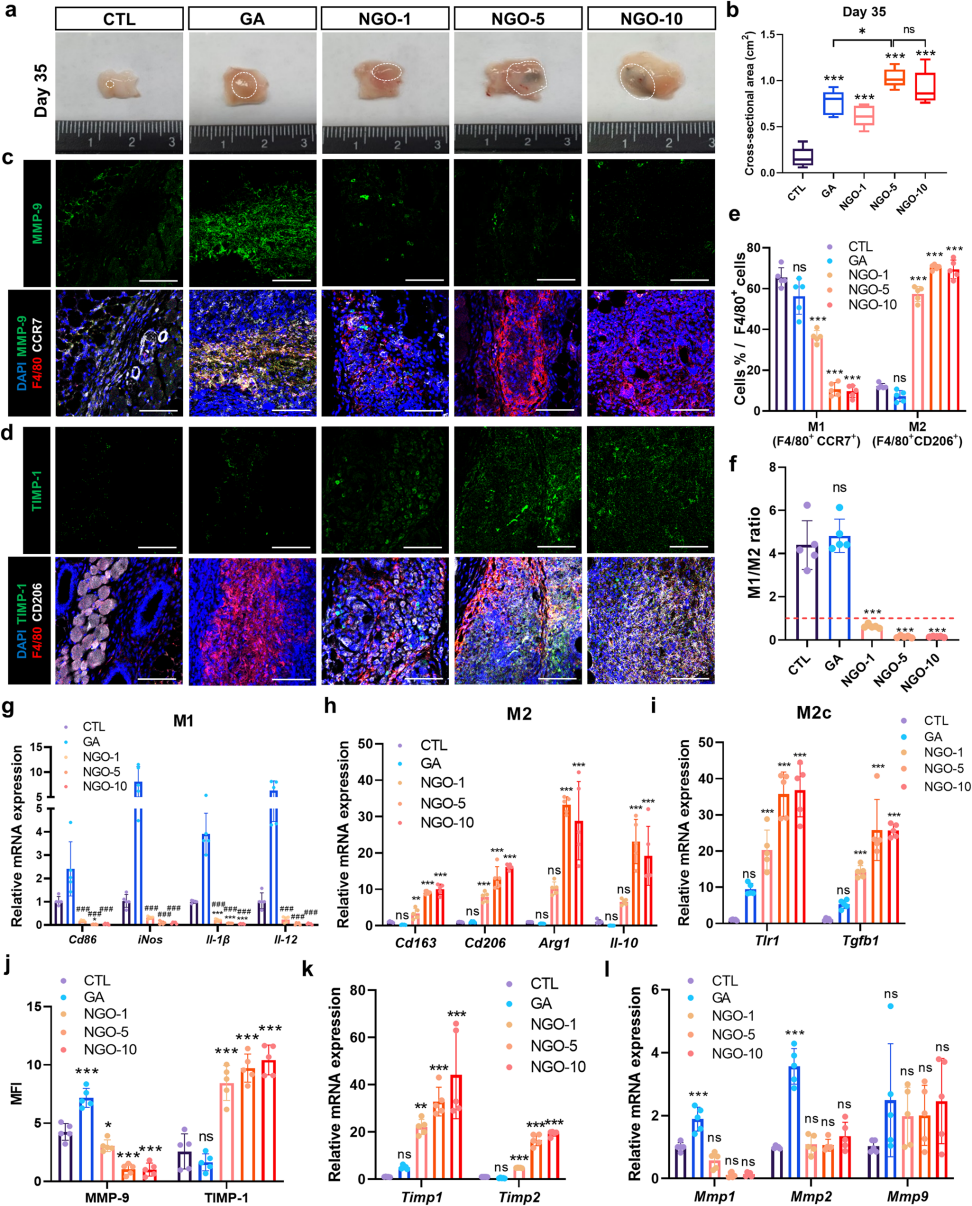
Constructive remodeling of NGO-crosslinked scaffolds by M2c macrophages. Gross images **(a)** of harvested scaffolds and the box plot **(b)** showing cross-sectional area of harvested scaffolds after 35 days of transplantation. In each box plot, the center line within the box indicates the median, the edges of the box indicate the 25th and 75th percentile, and the whiskers indicate outliers outside the 10th and 90th percentile (*n* = 5). Representative confocal images of the implants on day 35 in each group stained with the following antibodies; DAPI (blue), F4/80 (red), MMP-9 (green) and CCR7 (white) **(c)**, and TIMP-1 (green) and CD206 (white) **(d)**. Scale bar, 400 µm. **e** Quantification of infiltrating M1 and M2 macrophages normalized to F4/80^+^ cells within the implants (*n* = 5). **f** Ratio of M1^+^ cells to M2^+^ cells in each group (*n* = 5). qRT-PCR of M1 **(g)**, M2 **(h),** and M2c **(i)** -related genes in each scaffold harvested on day 35. (n = 5) **j** Mean fluorescence intensity (MFI) of MMP-9 and TIMP-1 in the stained sections of each group (*n* = 5). qRT-PCR analysis of mouse Timp subtypes **(k)** and Mmp subtypes **(l)** in each scaffold harvested on day 35 (*n* = 5). Quantitative data were presented as a mean ± SD. Statistical differences between the groups were determined by ordinary one-way ANOVA with post-hoc Tukey test (*p < 0.05, **p < 0.01, ***p < 0.001 versus CTL. ^###^*p* < 0.001 versus GA. ns; not statistically significant). Source data are provided as a Source Data file.

Upon scaffold transplantation, both mechanical strain leading to deformation of the material body and chemical stimuli including enzymes, cytokines and chemokines, may act in concert. In this regard, each crosslinked scaffold was transplanted subcutaneously to thoroughly evaluate the susceptibility to in vivo degradation. On day 7, the remaining area of the CTL scaffolds was significantly reduced compared with that of the scaffolds crosslinked with either NGO or GA (Supplementary Fig. 4a, b). On day 35, a considerable amount of scaffolds crosslinked with GA, NGO-5 and NGO-10 still remained, whereas the CTL scaffolds underwent drastic degradation (Fig. 4a, b). The scaffolds and their matricryptins are engulfed by phagocytes in the context of foreign body response, subsequently activate immune cells to release pro-inflammatory cytokines, and amplify further inflammation^11,16^. These responses can also stimulate MMP expression in the inflamed tissues, and the cascading effects, in turn, influence the ECM remodeling of the implants^33^. In this light, the systemic inflammatory responses in the recipient mice were evaluated by screening serum levels of pro-inflammatory cytokines and chemokines. There was no significant increase in pro-inflammatory cytokines in the NGO groups compared to those in the CTL group until day 7 and 35, in contrast to the GA group showing a clear manifestation of severe inflammation (Supplementary Fig. 4c, d). In particular, the serum levels of pro-inflammatory IL-12p70 and MCP-1 in the NGO groups on day 35 were markedly reduced compared to the CTL group (Supplementary Fig. 4e). More importantly, the local influx of neutrophils secreting proteases into the transplanted scaffolds, as the first line of defense, is strongly associated with ECM degradation and tissue-destructive functions^13^. Thus, we verified that the neutrophil infiltration within the transplanted scaffold and its vicinity was significantly reduced in the NGO-5 and NGO-10 groups, compared to the CTL and GA groups on day 7 and 35 (Supplementary Fig. 4f, g). As another part of innate immunity, differentiated M1-like or M2-like macrophages manage inflammatory responses in inflamed tissues. It has been well established that M1 macrophages participate in type 1 immunity and graft rejection, while M2 macrophages are implicated in type 2 immunity that inhibits type 1 inflammation and induces graft tolerance^34,35^. Notably, a remarkable accumulation of CD206^+^ or CD163^+^ M2-like macrophages, rather than CCR7^+^ or iNOS^+^ M1-like macrophages, was observed in the scaffolds crosslinked with NGO-5 and NGO-10 on day 7, and even until day 35 after transplantation (Fig. 4c–f, Supplementary Fig. 5a–e). Quantitative RT-PCR (qRT-PCR) analysis also demonstrated that the expression of M2-related genes in the scaffolds crosslinked with at least 5 μg/mL NGO was significantly upregulated, in contrast with that of M1-related genes (Fig. 4g, h). Subsequently, the adaptive immunity can be activated by the cues provided from the innate immunity, particularly macrophages^36^. Of note, as a critical component of adaptive immune responses, regulatory T (Treg) cells restrain the inflammation in the graft by inhibiting the entry of effector T cells into the graft, which have been identified as an important mechanism for inducing graft tolerance^37,38^. Hence, we verified that more CD4^+^ FOXP3^+^ Treg cells were recruited into transplanted NGO-crosslinked scaffolds compared to the CTL or GA scaffolds on day 35 (Supplementary Fig. 5f, g). qRT-PCR results also showed that the expression of genes, *Il-7R* and *Foxp3*, associated with graft tolerance was significantly increased in the NGO groups (Supplementary Fig. 5h). Taken together, NGO crosslinking could alleviate graft-elicited inflammation as evidenced by the reduction of neutrophil invasion, the predominance of immunoregulatory type 2 immune responses engaged with M2 macrophages, and enhanced infiltration of Treg cells.

### Constructive remodeling of NGO-crosslinked scaffolds via M2c polarization

Among several types of M2 macrophages, M2c macrophages are known to orchestrate matrix remodeling by releasing MMPs and TIMPs (tissue inhibitors of metalloproteinase)^15,39^. TIMP-1 is predominantly expressed by M2c macrophages, although some MMPs are also expressed in M2c macrophages^39^. In contrast, the proteolytic balance, a ratio of MMP-9 to TIMP-1, is known to be higher in M1 macrophages^39,40^. Thus, qRT-PCR analysis was performed to define a specific M2 subtype that occupied the implants. The results showed a significant upregulation of M2c-specific genes in the NGO-crosslinked scaffolds, reflecting that NGOs played a role in commitment of M2c macrophages (Fig. 4i). Given that the proteolytic balance determined the maintenance of ECM structures, the expression of MMP-9 and TIMP-1 in the implanted area was investigated. We observed M2-like cells strongly expressing TIMP-1 and a relative lack of catalytic MMP-9 expression in the NGO-5 and NGO-10 groups, whereas M1-like macrophages expressing MMP-9 were found mostly in the CTL and GA groups (Fig. 4c, d, j). In addition, high expression of TIMP-2 as well as low expression of catalytic MMP-2 in the NGO groups (Supplementary Fig. 6a, b). It was noteworthy that the mRNA expression of *Timp1* and *Timp2* were elevated in the NGO-5 and NGO-10 groups, compared to the CTL and GA groups. However, the transcript levels of several MMP subtypes in the NGO groups were not significantly reduced (Fig. 4k, l). Given that the protein expression of MMP catalytic domains was hardly detected in the NGO groups (Fig. 4c, Supplementary Fig. 6a, b), these combined results implicated that NGOs might directly interact with active forms of translated MMP proteins released from immune cells and suppressed their activities (Figs. 3, 4). Then, NGO itself and TIMPs released from M2c-like may contribute to MMP suppression simultaneously, thereby affecting the remodeling of dECM scaffolds in ways that were less degradable. As part of the constructive remodeling, the number of infiltrating α-SMA^+^ cells secreting ECM and the expression of ECM-related genes were elevated in the NGO groups (Supplementary Fig. 6c–e). Collectively, NGOs promoted a pro-reparative local milieu in the scaffolds by promoting M2c polarization and suppressing the expression of MMPs.

### Human M2c macrophage polarization within NGO-crosslinked scaffolds

**Fig. 5:**
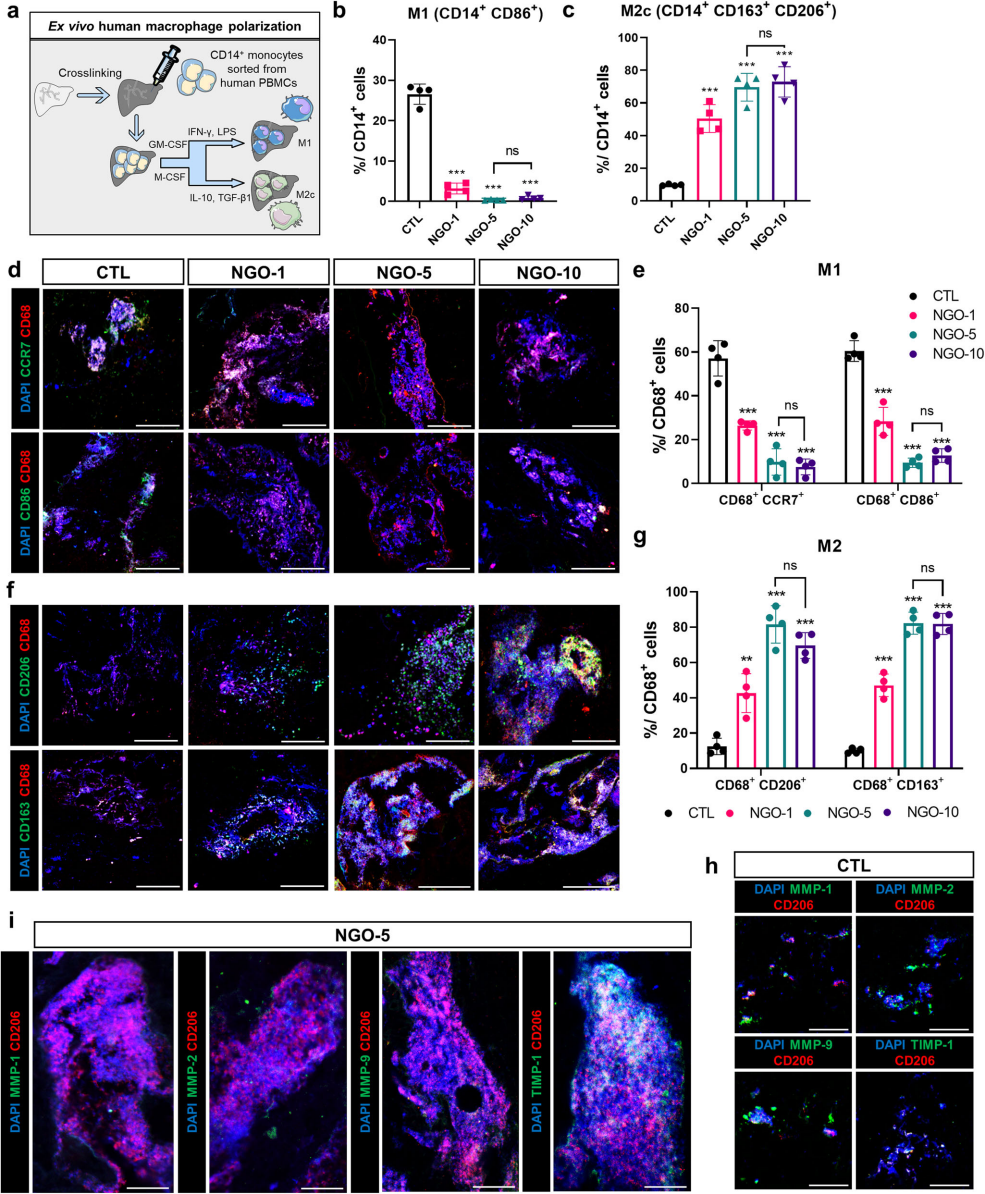
Ex vivo macrophage polarization from hPBMCs within NGO-crosslinked scaffolds. **a** Schematic diagram of ex vivo macrophage differentiation from human CD14^+^ monocytes within scaffolds crosslinked with nano-graphene oxide (NGO). Flow cytometry analysis of M1 **(b)** and M2c **(c)** expression within CD14^+^ populations in each group (*n* = 4). **d** Representative confocal images of M1 (CCR7, CD86: green, CD68: red, DAPI: blue). Scale bar, 100 µm. **e** Quantification of M1 normalized to CD68^+^ cells (*n* = 4). **f** Representative confocal images of M2 (CD206, CD163: green, CD68: red, DAPI: blue). Scale bar, 100 µm. **g** Quantification of M2 normalized to CD68^+^ cells (*n* = 4). Magnified confocal images of the non-crosslinked scaffolds (CTL) **(h)** and crosslinked scaffolds with 5 μg/mL of NGOs (NGO-5) **(i)**, Probed with CD206 (red), DAPI (blue) and following primary antibodies: MMP-1, MMP-2, MMP-9 and TIMP-1 (green). Scale bar, 40 µm. Quantitative data were presented as a mean ± SD. Statistical differences between the groups were determined by ordinary one-way ANOVA with post-hoc Tukey test (**p < 0.01, ***p < 0.001 versus CTL, ns; not statistically significant). Source data are provided as a Source Data file.

Based on the in vivo results we obtained, human macrophage differentiation was conducted ex vivo to predict whether NGOs could also affect macrophage polarization in humans. First, human peripheral blood-derived mononuclear cells (hPBMC)-derived CD14^+^ cells were differentiated into M1-like or M2c-like macrophages in response to respective cytokine stimuli within the scaffolds^41^ (Fig. 5a). Flow cytometric profiles clearly showed that NGO crosslinking repressed the shift to human M1-like phenotype expressing CD86 (Fig. 5b). In contrast, polarization into the human M2c-like macrophages expressing CD163 and CD206 was significantly enhanced particularly in the NGO-5 group (Fig. 5c). Similarly, the immunostaining results revealed that CD14^+^ monocytes seeded in the CTL scaffolds were dominantly differentiated to M1-like macrophages expressing CCR7 (Fig. 5d, e), whereas M2c differentiation was markedly promoted in the NGO-5 and NGO-10 groups (Fig. 5f, g). Based on these data, NGO-5 was regarded as an optimal environment for human macrophage polarization toward M2c. Therefore, we further confirmed that the expression of human MMPs (MMP-1, MMP-2 and MMP-9) were effectively inhibited, while a large number of human M2c-like macrophages expressing TIMP-1 were present within the scaffold crosslinked with NGO-5, compared to the CTL scaffolds (Fig. 5h, i). These results offered the possibility that degradation of implanted scaffolds and inflammatory responses elicited by transplantation in humans could be ameliorated in the presence of the NGOs promoting M2c polarization.

### Long-term maintenance of NGO-crosslinked scaffolds in vivo

**Fig. 6:**
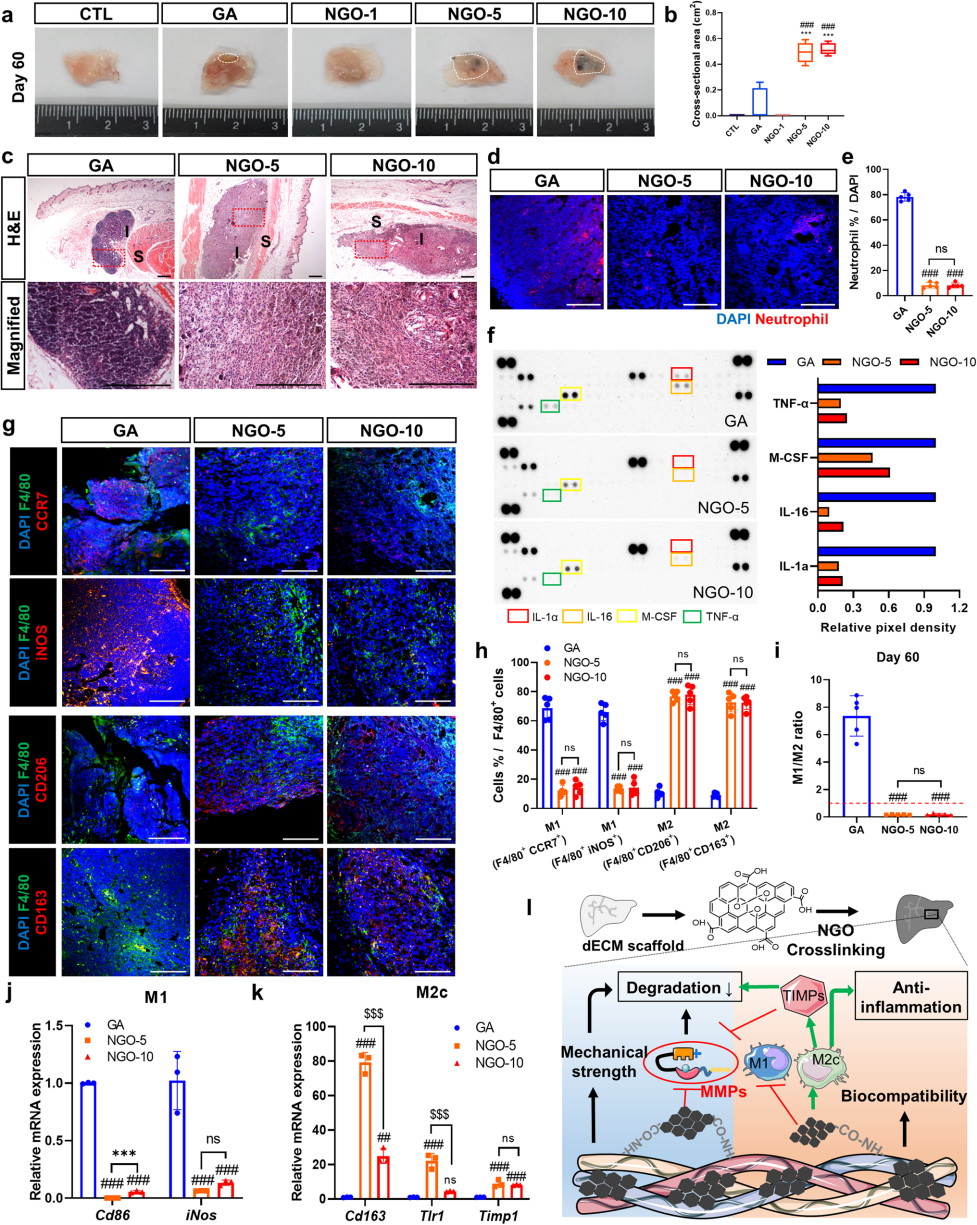
Evaluation of NGO-crosslinked scaffolds on day 60 after transplantation. Gross images **(a)** and the box plot **(b)** showing cross-sectional area of scaffold remnants harvested on day 60 in each group. In each box plot, the center line indicates the median, the edges of the box indicate the 25th and 75th percentile, and the whiskers indicate the 10th and 90th percentile (*n* = 5). **c** H&E staining of scaffold remnants on day 60 in each group (I: Implanted scaffolds, S: Surrounding tissues) and magnified images. Scale bar, 200 µm. **d** Representative confocal images of the implants probed with neutrophil (red) and DAPI (blue). Scale bar, 100 µm. **e** Quantification of infiltrating neutrophils in each group (*n* = 5). **f** Global cytokine analysis of the serum samples collected from the mice receiving each implant and sham mice on day 60, and quantification of pixel density of the spots corresponding to IL-1a, IL-16, M-CSF and TNF-α (*n* = 2). **g** Immunofluorescence analysis of the implants harvested on day 60. M1 (CCR7, iNOS; red), M2 (CD206, CD163; red) markers, pan-macrophage marker (F4/80; green), and DAPI (blue). Scale bar, 200 µm. **h** Quantification of detected M1 and M2 macrophages normalized to F4/80^+^ cells (*n* = 5). **i** Ratio of M1^+^ cells to M2^+^ cells within the implants (*n* = 5). qRT-PCR analysis of M1 **(j)** and M2c **(k)** -related genes in each group on day 60 (*n* = 3). **l** Schematic diagram of pivotal roles of nano-graphene oxide (NGO) in the events following transplantation of decellularized extracellular matrix (dECM) scaffolds. Quantitative data were presented as a mean ± SD. Statistical differences between the groups were determined by ordinary one-way ANOVA with post-hoc Tukey test (***p < 0.001 versus CTL, ^##^*p* < 0.01, ^###^*p* < 0.001 versus GA, ^$$$^*p* < 0.001, ns; not statistically significant). Source data are provided as a Source Data file.

To ensure the effects of NGOs on resisting scaffold decomposition, we maintained the transplants for 60 days. Notably, the scaffolds crosslinked with NGO-5 and NGO-10 remained highly stable after 60 days of implantation, whereas the scaffolds in the CTL and NGO-1 groups were completely absorbed (Fig. 6a, b). Compared to the GA group, we observed a significant reduction of the overall cellular infiltrates (Fig. 6c), as well as neutrophil infiltration (Fig. 6d, e) in the NGO-crosslinked scaffolds. In addition to local inflammation, low levels of systemic pro-inflammatory cytokines in the NGO-5 group were observed, compared to the GA groups (Fig. 6f). A favorable mononuclear cell response, predominantly shifting toward M2 rather than M1 macrophages, in the NGO groups was demonstrated by immunostaining (Fig. 6g, i) and qRT-PCR analysis (Fig. 6j, k). Taken together, type 2 inflammatory phenotypes and the predominance of M2c-like macrophages in the NGO groups may contribute to acquiring long-term resistance against inflammation-mediated degradation. Although the GA-crosslinked scaffolds partially remained until day 60, the intense infiltration of neutrophils and M1-like macrophages as well as systemic inflammation was observed. Collectively, NGO crosslinking had beneficial effects on the scaffold structural stability and the attenuation of tissue-destructive inflammation in the scaffolds following transplantation (Fig. 6l).

### Improved biocompatibility and functionality of human cells within NGO-crosslinked scaffolds

Prior to reconstruction of bioengineered livers, we assessed the cytotoxicity of crosslinked scaffolds using human chemically derived hepatic progenitors (hCdHs) and endothelial cells (ECs). MTT assay showed that the scaffolds crosslinked with NGOs possessed highly viable hepatocyte-like cells on day 15 (Supplementary Fig. 7a). Furthermore, the elevated secretion of albumin and urea in the NGO groups indicated the improved secretory functions of hepatocyte-like cells (Supplementary Fig. 7b, c). PAS staining also showed more accumulated glycogen (magenta granules) in the cells, suggesting the increased synthetic function of hepatocytes in the NGO-crosslinked scaffolds (Supplementary Fig. 7d). Similarly, the viability and vascular functions, including the secretion of human VEGF and nitric oxide, of the residing ECs were significantly enhanced in the NGO-5 and NGO-10 scaffolds (Supplementary Fig. 7e–g). These data suggested that the NGO-crosslinked scaffolds were highly biocompatible, hence, NGO-crosslinked scaffolds were considered adequate for fabrication of bioengineered livers. In contrast, despite the high capability of GA in strengthening scaffolds, GA crosslinking has limitations in its applications due to low biocompatibility.

### Reconstruction of MBLs using NGO-crosslinked scaffolds

**Fig. 7:**
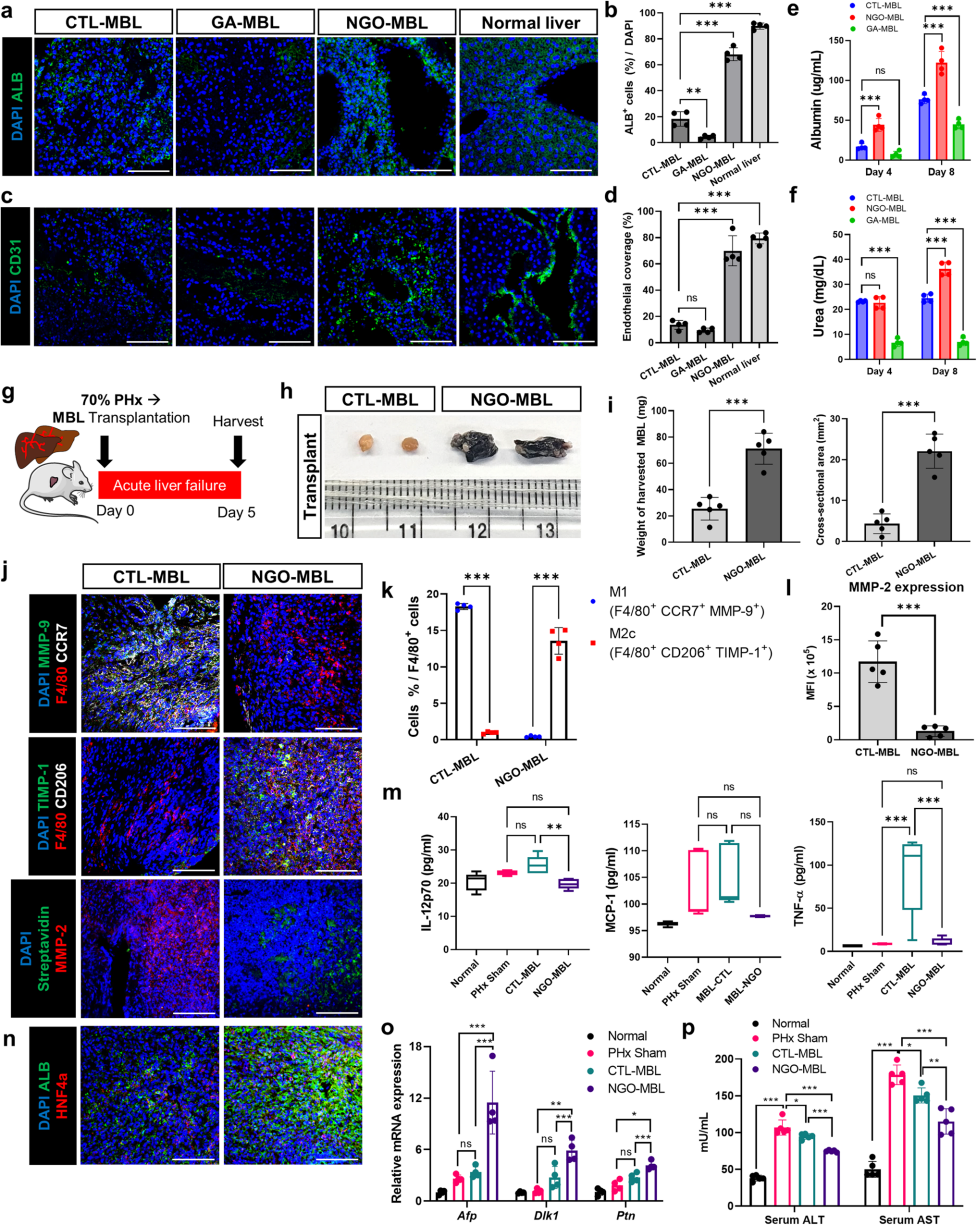
Transplantation of MBLs into a mouse model of acute liver failure. **a** Immunostaining of normal mouse livers and mouse bioengineered livers (MBLs) fabricated using scaffolds without crosslinking (CTL-MBLs), crosslinked with glutaraldehyde (GA-MBLs), and crosslinked with nano-graphene oxide (NGO-MBLs). ALB (green) and DAPI (nuclei, blue). Scale bar, 100 µm. **b** Quantification of ALB^+^ cells (*n* = 4). **c** Immunostaining of CD31 (green) and DAPI (nuclei, blue). Scale bar, 100 µm. **d** Quantification of CD31^+^ cells covering vascular lumen (*n* = 4). Quantification of secreted albumin **(e)** and urea **(f)** from CTL-MBLs, NGO-MBLs and GA-MBLs during bioreactor culture on day 4 and 8 (*n* = 4). **g** Schematic diagram of transplantation of MBLs into the mice receiving 70% partial hepatectomy. **h** Gross images of transplanted CTL-MBLs and NGO-MBLs on day 5. **i** Residual amounts of transplanted CTL-MBLs and NGO-MBLs, characterized by cross-sectional area and weight of transplants (*n* = 5). **j** Immunostaining of harvested CTL-MBLs and NGO-MBLs probed with following antibodies: F4/80 (red), DAPI (blue), MMP-9 (green) and CCR7 (white), TIMP-1 (green) and CD206 (white), streptavidin (green) and MMP-2 (red). Scale bar, 100 µm. Quantification of M1 macrophages and M2 macrophages normalized to F4/80^+^ cells **(k)**, and MMP-2 expression **(l)** within CTL-MBLs and NGO-MBLs (*n* = 5). **m** Cytometric bead array of serum samples from the following groups; Normal mice, the mice receiving partial hepatectomy (PHx Sham), the mice receiving CTL-MBL and NGO-MBL transplantation after PHx. In each box plot, the center line indicates the median, the edges of the box indicate the 25th and 75th percentile, and the whiskers indicate the 10th and 90th percentile (*n* = 5). **n** Transplanted CTL-MBLs and NGO-MBLs stained with ALB (green) and HNF4α (red). Scale bar, 100 µm. **o** qRT-PCR analysis of liver regeneration-related genes (*n* = 5). **p** Serum levels of ALT and AST in each mouse on day 5 (*n* = 5). Quantitative data were presented as a mean ± SD. Statistical differences between the groups were determined by ordinary one-way ANOVA with post-hoc Tukey test (**b**, **d**–**f**, **m**, **o**, **p**) and unpaired, two-tailed student’s *t* test (**i**, **k**, **l**) (*p < 0.05, **p < 0.01, ***p < 0.001, ns; not statistically significant). Source data are provided as a Source Data file.

To address whether NGO-crosslinked scaffolds are beneficial for the reconstruction of bioengineered livers, we fabricated allograft MBLs by using mouse-induced hepatocytes (miHeps) and mouse endothelial cells (mECs) (Supplementary Fig. 8a). It is known that both the physical properties of scaffolds and graphene itself can affect cell differentiation and functions^42,43^. Thus, we first investigated whether the NGOs could influence hepatic differentiation and functionalities of miHeps. We identified that the expression of hepatocyte markers (ALB and CK18) of miHeps was increased after maturation in the NGO-5 and NGO-10 scaffold discs (Supplementary Fig. 8b, c). Furthermore, the secretion of albumin and urea from the miHep-seeded scaffold discs was also significantly elevated in the NGO groups (Supplementary Fig. 8d, e). Thus, the NGO-5 and NGO-10 scaffolds were considered the most effective condition for augmenting the differentiation and hepatic functions of the engrafted cells. The mixture of miHep maturation medium and mEC culture medium at a ratio of 2:1 was optimal to maintain both characteristics during co-culture (Supplementary Fig. 8f). To fabricate MBLs with crosslinked scaffolds, miHeps and mECs were seeded into non-crosslinked scaffolds (CTL-MBL), NGO-crosslinked scaffolds (NGO-MBL) and GA-crosslinked scaffolds (GA-MBL), and maintained for 10 days (Supplementary Fig. 8g). Notably, the NGO-MBLs resembled normal liver structures more closely than the CTL-MBLs and GA-MBLs in terms of the distribution of ALB^+^ parenchymal cells (Fig. 7a, b). Moreover, CD31^+^ ECs in the NGO-MBLs aligned along the vascular lumen of the scaffolds, whereas the CTL-MBLs showed fewer CD31^+^ ECs around the vasculature (Fig. 7c, d). The GA-MBLs possessed few functional cells because of the high apoptotic rate, as evidenced by high expression of cleaved Caspase-3 (Fig. 7a–d, Supplementary Fig. 8h, i). In addition to histologic similarities, liver-specific functions were also enhanced in the NGO-MBLs, compared to the CTL-MBLs (Fig. 7e, f). Collectively, NGO crosslinking enabled the reconstruction of MBLs with elaborate histological structures and improved functionalities.

### Effective regeneration of dysfunctional livers via transplantation of NGO-MBLs into liver failure mouse model

**Fig. 8:**
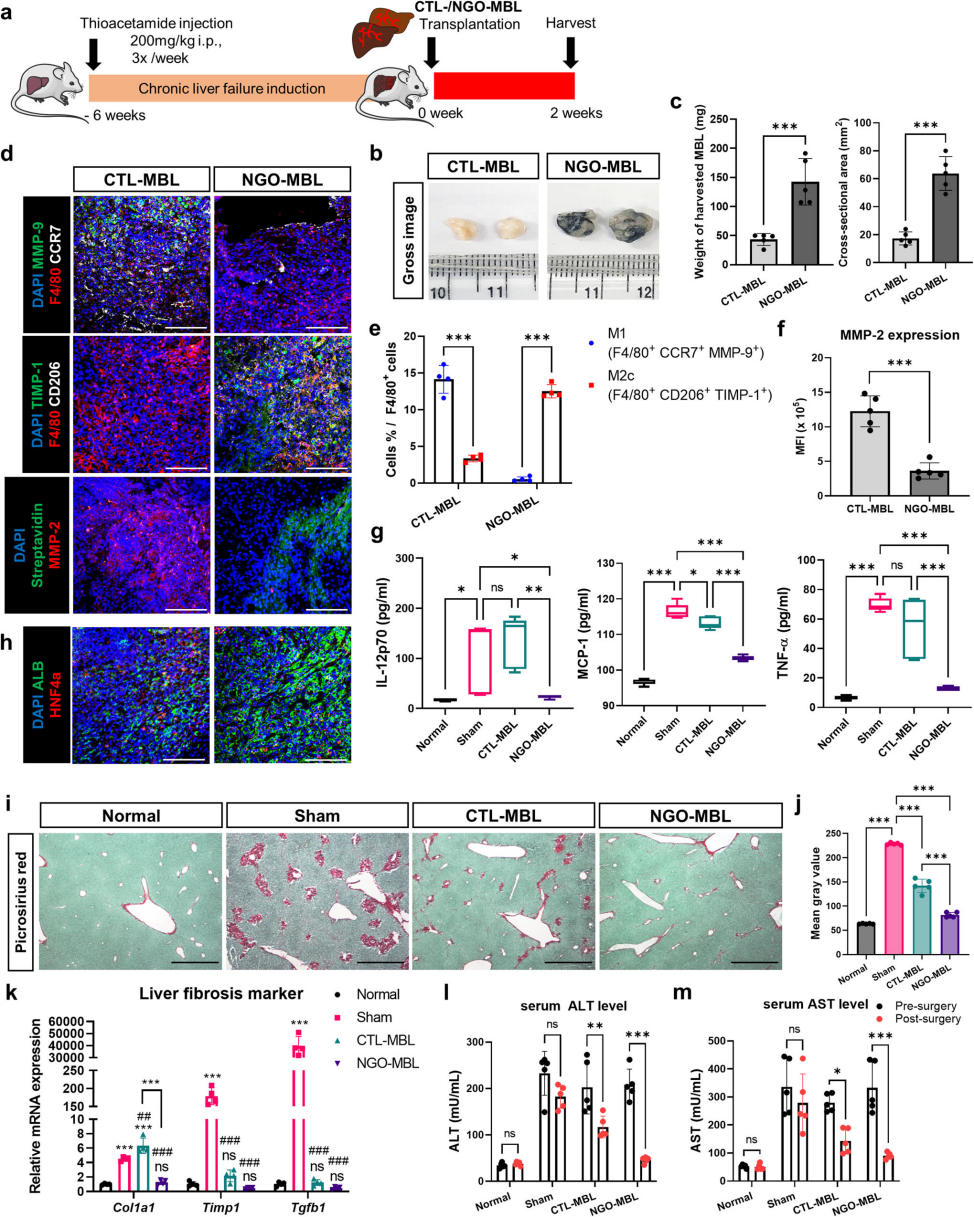
Transplantation of MBLs into a mouse model of chronic liver failure. **a** Schematic diagram of the strategy for establishing thioacetamide (TAA)-induced chronic liver failure model and subsequent transplantation of mouse bioengineered livers produced by using scaffolds without crosslinking (CTL-MBLs) and crosslinked with nano-graphene oxide (NGO-MBLs). **b** Gross images of remaining CTL-MBLs and NGO-MBLs at 2 weeks after transplantation. **c** The residual amounts of CTL-MBLs and NGO-MBLs, characterized by cross-sectional area and weight of residual transplants (*n* = 5). **d** Immunostaining of harvested CTL-MBLs and NGO-MBLs probed with following antibodies: F4/80 (red), DAPI (blue), MMP-9 (green) and CCR7 (white), TIMP-1 (green) and CD206 (white), and streptavidin (green) and MMP-2 (red). Scale bar, 100 µm. Quantification of M1 macrophages and M2 macrophages normalized to F4/80^+^ cells **(e)**, and MMP-2 expression **(f)** in each group (*n* = 5). **g** Cytometric bead array performed with the serum collected at 2 weeks from the normal mice, the mice in which chronic liver injury was induced (Sham), the mice receiving CTL-MBL and NGO-MBL transplantation. In each box plot, the center line indicates the median, the edges of the box indicate the 25th and 75th percentile, and the whiskers indicate the 10th and 90th percentile (*n* = 5). **h** Immunostaining of harvested CTL-MBLs and NGO-MBLs. ALB (green), HNF4α (red) and DAPI (blue). Scale bar, 100 µm. Picrosirius red staining **(i)** of livers in each group and quantification **(j)** of fibrotic area. Scale bar, 40 µm (*n* = 5). **k** qRT-PCR analysis of liver fibrosis-related genes in each group (*n* = 5). Serum ALT **(l)** and AST **(m)** levels of mice in each group, before and after transplantation (*n* = 5). Quantitative data were presented as a mean ± SD. Statistical differences between the groups were determined by unpaired, two-tailed student’s *t* test (**c**, **e**, **f**), Ordinary one-way ANOVA with post-hoc Tukey test (**g**, **j**, **k**) and two-way ANOVA with post-hoc Tukey test (**l**, **m**) (*p < 0.05, **p < 0.01, ***p < 0.001 versus CTL-MBL, ^##^*p* < 0.05, ^###^*p* < 0.001 versus Sham, ns; not statistically significant). Source data are provided as a Source Data file.

To evaluate the in vivo durability and consequential regenerative potential of MBLs, we established acute and chronic liver failure mouse models. We first surgically induced acute liver failure by performing 70% partial hepatectomy (70% PHx) in male BALB/c mice (Supplementary Fig. 9a). A drastic reduction of liver mass-induced hepatic dysfunction as evidenced by elevated ALT and AST serum levels (Supplementary Fig. 9b, c). Then, the CTL-MBLs and NGO-MBLs were transplanted and harvested on day 5 (Fig. 7g). As the MBLs were gradually degraded upon transplantation, the residual amount of NGO-MBLs was larger than that of CTL-MBLs on day 5 (Fig. 7h, i). Of note, M2-like macrophages expressing TIMP-1 predominantly accumulated within the transplanted NGO-MBLs while the expression of MMP-9 and MMP-2 was markedly suppressed in the presence of NGOs (Fig. 7j–l). Of note, activation of hepatic stellate cells (HSCs) has also been deeply linked to ECM synthesis and remodeling^44^. However, there was no significant difference in the amount of activated HSCs infiltrated in CTL-MBLs and NGO-MBLs (Supplementary Fig. 9d, e). Considering that inflamed transplants trigger systemic inflammation, increased serum levels of pro-inflammatory cytokines and spleen enlargement were demonstrated in the CTL-MBL group while these effects were alleviated with NGO-MBL transplantation (Fig. 7m, Supplementary Fig. 9f, g). In addition, the NGO-MBLs not only contained a substantial amount of remaining scaffold but also numerous ALB^+^ HNF4α^+^ hepatocyte-like cells within the graft, in contrast to the CTL-MBLs (Fig. 7n). Hence, we next investigated whether NGO-MBL transplantation could promote the regeneration efficiency of the recipient liver. Given that LGR5^+^ cells were highly proliferative hepatic stem cells that regulated liver regeneration^45^, more LGR5^+^ cells were populated within the NGO-MBL transplants, suggesting the promoted regeneration of the injured livers (Supplementary Fig. 9h, i). It is worth noting that the mRNA expression of genes associated with hepatic regeneration was upregulated in the livers receiving NGO-MBL transplantation (Fig. 7o). Moreover, serum ALT and AST levels were significantly decreased in the NGO-MBL group compared with those in the CTL-MBL group (Fig. 7p). Altogether, the NGO-MBLs protected the scaffolds against degradation and maintained engrafted cell populations, and eventually exhibited a superior regenerative potential compared to the CTL-MBLs. Next, we explored the long-term regenerative capability of the MBLs by transplantation into a thioacetamide (TAA)-induced chronic liver failure model (Fig. 8a, Supplementary Fig. 10a). The remaining amounts of the NGO-MBLs were higher than those of the CTL-MBLs at 2 weeks after transplantation (Fig. 8b, c). In the NGO-MBL transplants, a robust influx of M2-like macrophages expressing TIMP-1 was observed while MMP-9 and MMP-2 were expressed at low levels, suggesting that the NGOs contributed to ECM protection by regulating the proteolytic balance within the transplants (Fig. 8d–f). We also noted the possibility of fibrosis of transplanted MBLs on day 14 due to M2-governing type 2 immune responses^35^. However, the excessive ECM accumulation in the NGO-MBL transplants was not observed compared to the CTL-MBL transplants and the fibrotic livers (Supplementary Fig. 10b). Furthermore, the proportion of infiltrating activated HSCs was not significantly different between the two groups, suggesting that the effects of NGO crosslinking on ECM remodeling in transplanted MBLs was not mediated by activated HSCs (Supplementary Fig. 10c, d). Furthermore, NGO-MBL transplantation alleviated the systemic inflammation in the recipients as demonstrated by reductions in serum pro-inflammatory cytokines and spleen size, in contrast to CTL-MBL transplantation (Fig. 8g, Supplementary Fig. 10e, f). More importantly, transplanted NGO-MBLs retained a number of functional hepatocyte-like cells expressing ALB and HNF4α (Fig. 8h). Accordingly, the recipient livers receiving NGO-MBL transplantation displayed less fibrotic lesions (Fig. 8i, j) and a significant reduction of fibrosis-related gene expression (Fig. 8k), both indicating the high regenerative potential of the NGO-MBLs. Serum ALT and AST levels, which had been increased by TAA induction, were considerably decreased after the transplantation of the NGO-MBLs, suggesting the functional restoration of the damaged livers (Fig. 8l, m). In summary, due to the pro-regenerative niche created by NGOs, the NGO-MBLs effectively carried out hepatic regeneration for long-term than CTL-MBLs when transplanted into liver failure mouse models.

## Discussion

As a viable alternative to living donor organs for liver transplantation, bioengineered livers have been constructed with dECM scaffolds that retain a biomimetic 3D microenvironment^8^. However, liver dECM is vulnerable to enzymatic destruction caused by graft-elicited inflammation that occurs because of large interspecific incompatibility, as well as mechanical deformation, after transplantation. Thereby, these factors detrimentally affected graft survival. Hence, there has been a continuous need for a strategy for reinforcing the mechanical properties of the scaffolds and simultaneously ameliorating inflammatory responses following transplantation. In this study, NGO was utilized as a biocompatible crosslinking agent for dECM scaffolds and reconstruction of bioengineered livers, which has not been explored yet.

NGOs are advantageous for participating in reactions with ECM fibers during crosslinking owing to abundant oxygen-containing functional groups and their small size. Through characterization by various methodologies, it was shown that NGOs possessed oxygen-containing functional groups, such as carboxylic acid, hydroxyl, epoxide and carbonyl groups. Our ATR-FTIR results indicated that the free primary amine groups in the dECM scaffolds primarily reacted with the carboxylic acid groups and partially with the epoxy rings of the NGOs, forming amide bonds. As previously reported^28^, carboxyl groups are mainly located at the edges of graphene sheets, while epoxy groups are located in the basal plane. Hence, the carboxyl groups on the edge have the highest reactivity for forming covalent bonds with the molecules in other functional groups^46^. Theoretically, the carbonyl groups that are randomly spread across edges and the basal planes of the graphene can also react with primary amine groups and form imines^47^. However, imine peaks (1610–1622 cm^−1^) were not detected in FTIR spectra of the NGO groups, suggesting that the reactivity of carbonyl groups is lower than that of other functional groups. Another distinctive feature of NGOs would be their small size. NGOs are likely to be more reactive than large GOs because they easily penetrate into ECM fibrils and have more functional groups at the edges due to their high edge-to-area ratio^48^. Although GOs tend to aggregate due to negative charge and van der Waals forces^49,50^, nanosized GOs have a high salt tolerance and weakened van der Waals forces between layers, resulting in a greater dispersion stability and lower aggregation compared to microsized GOs^51^.

After NGOs are integrated into the scaffolds, the primary role of the NGOs is that of a highly effective crosslinker that improves the physical properties of ECM fibers to withstand mechanical or chemical stresses. Via NGO crosslinking, we successfully achieved a high resistance of the scaffolds against mechanical transformation, which was comparable to that of GA. Next, the chemical resistance of the NGO-crosslinked scaffolds against enzyme-mediated disruption was demonstrated by treatment with MMPs that primarily control the proteolysis of the ECM components upon transplantation^12,52^. Here, we also found that the NGOs showed inhibitory effects on MMP catalysis by directly interacting with the conserved regions of MMP catalytic domains. Some possible mechanisms underlying how the NGOs affected the catalytic activities of MMPs are proposed as follows. First, the glutamate residues in the catalytic domains of MMPs participate in nucleophilic attacks on the carbonyl carbons of the substrate for peptide cleavage^30^. Therefore, there is a possibility that the NGOs bound to glutamate residues may alter the proton transfer process after nucleophilic attack due to the proton dissociation of the oxygen-containing functional groups of the NGOs^53^, resulting in the catalytic dysfunction of the MMPs. Second, as histidine residues support catalytic zinc for structural stability^30^, the NGOs bound to histidine residues may cause 3D conformational changes and destroy the spatial control of zinc, thereby leading to the catalytic dysfunction. To further investigate the effects of NGOs on the electric and topographic features of MMPs, computational approaches, such as Molecular Dynamics (MD) simulation, can be valuable^54^. In this way, NGO crosslinking fundamentally improved the mechanical strength of the dECM scaffolds and protected the scaffolds from proteolysis.

Upon transplantation, inflammatory responses are strongly linked to the MMP-mediated degradation of the scaffolds. As xenograft-derived dECM scaffolds are recognized as foreign bodies by the host immune system, immune cells infiltrate into the transplants and release proteases to degrade the scaffolds^14,16^. Produced bioactive matricryptins not only stimulate systemic inflammation after being engulfed by phagocytes but also accelerate chemotaxis toward the transplants^11,16^. Thus, we investigated the inflammatory phenotypes after scaffold implantation from various perspectives; the local infiltration of neutrophils and Treg cells, polarization of infiltrating macrophages, and systemic inflammation. In combination with fragile ECM structures of non-crosslinked scaffolds, robust infiltration of highly proteolytic M1 macrophages and neutrophils amplified such tissue-damaging inflammatory responses, leading to an uncontrolled breakdown of the CTL scaffolds. In contrast, we provided in vivo evidence for the distinct effects of NGO crosslinking in which the NGO-crosslinked scaffolds were less prone to degradation until 60 days after transplantation. Based on the obtained results, the versatile roles of NGOs in mitigating ECM degradation were proposed as follows: i) the reinforcement of mechanical properties of the scaffold, ii) the improved biocompatibility of the scaffold itself, iii) the regulation of both immune milieu toward type 2 inflammation and graft tolerance, iv) the constructive remodeling of the scaffolds via polarization of the infiltrating macrophages toward M2c-like macrophages secreting TIMPs, and v) the direct inhibition of released MMPs. These effects listed above may comprehensively affect the inflammatory responses and matrix remodeling of the transplants^40,55^.

Through a series of experiments, we revealed that the concentration which fully exerts beneficial effects of NGOs was at least 5 μg/mL on crosslinking, biocompatibility and immune modulation of dECM scaffolds. In addition, the leveling-off of the NGO effects above 5 μg/mL was observed, which may arise from graphene aggregation. Even if 10 μg/mL of NGOs were used for crosslinking, the entire amount of injected NGO might not bind to the dECM scaffolds, and some NGOs remained free within the scaffolds. And these excessive amounts of free NGOs may cause adverse effects on the cells engrafted into the scaffolds. For instance, it has been reported that the cytotoxicity of excessively absorbed NGOs is manifested by interacting with cell membranes or producing reactive oxygen species, possibly resulting in injuries to lungs, kidneys, brain and reproductive systems^56^. Since an optimal NGO concentration may differ depending on the size and reactivity of NGOs, the issues regarding aggregation within the scaffolds, the reactivity, effectiveness and crosslinking efficiency of NGOs should be further investigated.

With the NGO-crosslinked scaffolds, MBLs were produced by seeding miHeps and mECs into crosslinked scaffolds. Since the NGO-MBLs exhibited more cells expressing ALB in the parenchymal region and CD31 in the vascular luminal surface, they closely resembled normal liver structures compared to the CTL-MBLs and GA-MBLs. However, some limitations should be considered to develop bioengineered livers with ideal structures and biochemical properties in the future: i) co-culture of more kinds of liver-composing cells, such as biliary epithelial cells, Kupffer cells and hepatic stellate cells, and ii) further protocol optimization depending on the injecting cell types, the total number of injected cells, the injected ratio between different cell types, and ex vivo culture duration. Liver grafts may function as a replacement to recipient livers to support liver functions, but at the same time, also a stimulator for liver regeneration in a paracrine manner. In this context, we transplanted MBLs into acute and chronic liver failure mouse models respectively, to validate the in vivo regenerative capacity of MBLs. Considering that the residual liver lobes grow in size within 1 week after 70% PHx in mice^57^, the mice were sacrificed on day 5 after transplantation of MBLs into an acute liver failure model to compare the regeneration before the recipient livers fully recovered from injuries. When the MBLs were transplanted into a chronic hepatic failure model, the mice were harvested after 2 weeks to give sufficient time for host macrophages to shift as previously described^58^. In both models, transplanted NGO-MBLs exhibited dominant accumulation of M2c-like macrophages expressing TIMP-1, which may help the implants to remain relatively intact. These constructive phenotypes with type 2 immune responses mediated by the NGOs contributed to the retention of more functional cell populations in the NGO-MBLs and resulted in a high regenerative capacity, compared to the CTL-MBLs. To extensively study the exact in vivo roles of macrophages in MBL transplantation, an animal model of macrophage depletion, either genetically or chemically-induced^59,60^, can be utilized in the future. In general, type 2 immunity implicated with M2 macrophages, has been associated with not only tissue repair but also tumor-permissive environments. Nonetheless, Wolf et al. reported that M2-biased microenvironment created by ECM materials showed tumor-suppressive effects because the phenotypes of M2 macrophages in the dECM niche were distinct from those of traditional M2 macrophages^55^. Considering that the functional diversity of macrophages during polarization have recently been reported^36,61^, our arguments need to be further validated by using single-cell phenotypic analysis, which will allow us to delineate the heterogeneity of infiltrating macrophages and predict possible tumorigenic risks in the transplants. Moreover, although we partly dispelled the possibility that NGOs might accelerate fibrosis of MBL transplants (Supplementary Fig. 10b), further investigation will be necessary to closely monitor the long-term effects of microenvironment created in the MBL transplants, including immune profiles, particularly for tumor immunosurveillance, over the long period.

In conclusion, we successfully established a highly durable, biocompatible and immunomodulatory scaffold through NGO crosslinking. We also highlighted the importance of NGO crosslinking in that less degradable NGO-MBLs had superior regenerative potential. However, it is indispensable to extend this study to a scale which is clinically applicable to humans. The porcine livers, which are the most similar in size to human livers, have been particularly utilized for clinical relevance. Considering that even porcine dECM scaffolds act as a xenograft and elicit a host immune response upon transplantation to humans, it is worthwhile to verify the in vivo beneficial roles of NGO crosslinking before scaling up the scaffold size. Although more in-depth investigations of the optimized crosslinking condition for porcine scaffold as well as the biochemical effects on the engrafted cells or recipient after transplantation are still needed, this study lays the cornerstone for creating transplantable human-sized bioengineered livers and moreover, other organs.

## Methods

### NGOs preparation and analysis

NGOs were readily synthesized from the graphite via Taylor-Couette flow^62^. The morphology of synthesized NGO particles was analyzed with Cs corrected HRTEM (JEM-ARM200F, Cold FEG, JEOL Ltd, Japan) after loaded to a 400 mesh carbon-coated copper grid. The size distribution of NGOs was analyzed using a CPS Disc Centrifuge (CPS instruments, USA). Next, NGOs prepared on a sapphire wafer were scanned by atomic force microscopy in noncontact mode (scanned area 25 μm^2^, XE-100, Park Systems, Republic of Korea). X-ray diffraction pattern of NGOs was obtained using SmartLab (Rigaku, Japan). The Raman spectrum of NGOs was obtained using 532 nm excitation laser (LabRAM HR Evolution, HORIBA, Japan). The powder of NGOs was subjected to X-ray photoelectron spectroscope (AXIS-His, Kratos, USA). FTIR spectrum of NGO was obtained by using FTIR Spectrophotometer (Nicolet 6700, Thermo Fisher Scientific, USA) with conventional KBr pellet method. A zeta potential of NGOs was analyzed using Zetasizer Nano ZSP (Malvern Instruments, England). For detecting NGOs, biotin (#14400, Sigma, USA) was conjugated to NGOs by EDC (#77149, Thermo Fisher Scientific) - NHS (#24525, Thermo Fisher Scientific) coupling.

### Fabrication of decellularized liver scaffolds and characterization

All rat experiments were performed in accordance with the approved guidelines of the Seoul National University Institutional Animal Care and Use Committee (SNU-191208-1). For fabrication of decellularized rat liver scaffolds, rat livers were collected from female Sprague Dawley rats (200–250 g), decellularized by perfusion of 0.1% sodium dodecyl sulfate via portal vein, and sterilized with 0.1% peracetic acid (Sigma), as previously established^9,63^. The fixated scaffolds were subjected to histological examination and SEM. DNA was extracted from the samples using AccuPrep Genomic DNA extraction kit (Bioneer, Republic of Korea). The concentration of DNA was measured by a spectrophotometer (Nanodrop 2000c, Thermo Fisher Scientific) and normalized to dry weight of each sample. To thoroughly investigate the proteomic profiles of the liver tissues, native liver and dECM liver was subjected to LC/MS analysis (Liquid Chromatography Hybrid-FT orbitrap Mass Spectrometer, Korea Basic Science Institute, Ochang, Republic of Korea). Rattus norvegicus database was downloaded from Uniprot (released on 2017/12/20: 31,571 sequences and 17,286,506 residues) and peptide-spectrum match step was performed under the following condition: 10 ppm of peptide mass tolerance and 0.8 Da of fragment mass tolerance. Eventually, the peptides and proteins below 1% of false discovery rate were sorted for each group. The iBAQ value for each protein was obtained by using MaxQuant. Using in silico approach, matrisome proteins in the native liver and dECM liver were sorted from LC/MS data using MatrisomeDB 2.0 (http://matrisomeproject.mit.edu/proteins/) according to previous report^64^. Based on the subdivided classifications, the matrisomes were categorized into the core matrisomes (collagens, ECM glycoproteins and proteoglycans) and the matrisome-associated proteins (ECM regulators, ECM-affiliated proteins and secreted factors). Top 30 proteins in core matrisome of native liver and dECM liver was sorted and their proportions to total core matrisomes were visualized using a heatmap (GraphPad).

### Crosslinking with NGOs

For crosslinking dECM scaffolds, 50 mL of each crosslinking reagent (PBS; negative control, 0.625% glutaraldehyde, 1 μg/mL, 5 μg/mL and 10 μg/mL of NGOs) was perfused via both bile duct and portal vein of liver scaffolds for 24 h at 4 °C. The crosslinked scaffolds discs (*r* = 6 mm) were fabricated using skin biopsy punch (P1250, Acuderm Inc., USA). To visualize the distribution of NGOs within the dECM scaffolds and determine the efficiency of NGO incorporation, biotinylated NGOs were perfused into the scaffolds and probed with fluorescent streptavidin. For characterization, lyophilized powers of crosslinked scaffolds were subjected to ATR-FTIR analysis (VERTEX800v, Bruker, USA). Raman spectra of the whole dried scaffold discs were recorded by using 532 nm laser. The weight of scaffold discs was measured before and after drying overnight and the swelling ratio of each scaffold was calculated as below.

Swelling ratio (%) = (*W*_s_ – *W*_d_) / *W*_d_ X 100 (*W*_s_: Swollen weight, *W*_d_: Dried weight)

### In vitro MMP resistance test

To mimic in vitro MMP-mediated biodegradation of the scaffolds, we used active MMP-1 (Sigma), recombinant MMP-2 (#420-02, Peprotech, USA) and recombinant MMP-9 (ab155704, Abcam) that were activated with aminophenylmercuric acetate for 1 h and 2 h respectively at 37 °C to cleave pro-peptides. Each scaffold was incubated with 100 μg of MMPs in 0.1 M Tris-HCl buffer (pH 7.6) for 2 h at 37 °C. The reaction was stopped by adding 10 mM EDTA solution (Gibco, USA). The weight loss during biodegradation was calculated by measuring the scaffold weight before and after the treatment of MMPs. MMP-treated scaffolds in each group were also subjected to ninhydrin assay and insoluble collagen quantification.

### In vivo transplantation of the crosslinked scaffolds

The experiments were conducted with the approval of Seoul National University Institutional Animal Care and Use Committee (SNU-191208-1). Sterilized scaffold discs in each group were placed in dorsal subcutaneous pockets of 6-week-old male BALB/c mice and harvested at the indicated time points (day 7, day 35 and day 60). For investigating the immune responses after implantation, infiltrating neutrophils, macrophages, and Treg cells within the implanted scaffolds were quantified using immunofluorescence analysis and qRT-PCR analysis. The serum samples harvested from the mice were subjected to global cytokine analysis for analyzing systemic inflammatory responses.

### Ex vivo macrophage polarization from hPBMCs

hPBMCs (CC-2702, Lonza, Switzerland) were cultured in RPMI 1640 (Gibco) supplemented with 10% FBS (fetal bovine serum, Gibco) for 24 h and then subjected to magnetic activated cell sorting using CD14 microbeads (130-050-201, Miltenyi Biotec, Germany). Then, 2 × 10^5^ of CD14^+^ cells were seeded into sterilized crosslinked discs in each group. After cell seeding, scaffolds were incubated with basal medium supplemented with 20 ng/mL GM-CSF for M1 and 20 ng/mL M-CSF for M2 for 2 days. For M1 polarization, 20 ng/mL of IFN-γ (Peprotech), 1 μg/mL of LPS (InvivoGen, USA) were added to the medium, and the scaffolds were maintained for an additional 5 days. For M2c polarization, scaffolds were additionally supplemented with 20 ng/mL of IL-10 (Peprotech) and 20 ng/mL of TGF-β1 (Peprotech) and maintained for 5 days. On day 7, the scaffolds were digested for flow cytometry analysis or fixated for immunostaining.

### Biocompatibility of the crosslinked scaffolds

The hCdHs were kindly provided by Dr. Dongho Choi (Hanyang University, Republic of Korea) and maintained as previously reported^65^. ECs were purchased from ATCC (USA) and maintained in endothelial growth medium-2 (Lonza) with 10% FBS in a 5% CO_2_ incubator at 37 °C. All culture media includes antibiotics (100 U/mL Penicillin and 100 μg/mL Streptomycin, Gibco). For assessing the biocompatibility of the crosslinked scaffolds, 5 × 10^5^ of hCdH cells and 2 × 10^5^ of ECs were seeded into each crosslinked scaffold disc respectively. After 2 h of static culture, cell-seeded scaffolds were maintained in hepatic differentiation media and endothelial media, respectively, for 15 days. Each scaffold at the indicated time points was subjected to further analysis.

### MBL generation and transplantation to liver failure mouse model

All experiments were conducted with the approval of Seoul National University Institutional Animal Care and Use Committee (SNU-211130-2). miHeps were cultured in miHep maintenance medium as previously reported^66^. Mouse primary ECs were purchased from Cell Biologics (#BALB-5017, USA) and cultured in EGM-2 (Lonza) supplemented with 10% FBS. To optimize the culture condition in which both miHeps and mECs maintain their characteristics, two types of cells were co-cultured by using transwell, followed by immunofluorescence staining. To fabricate the MBLs, 6 × 10^7^ of miHeps were seeded into the bile duct of CTL scaffolds and NGO-crosslinked scaffolds respectively. For a 3D culture, MBLs maintained in a bioreactor and were perfused with miHep maintenance medium by using a peristaltic pump for 2 days. On day 2, 2 × 10^7^ of mECs were seeded into the portal vein of each scaffold and 2:1 (miHep maturation medium: EC medium) medium was perfused for 7 days. On day 7, each MBL was transplanted into mouse models of acute or chronic liver failure. The male mice were used to preclude the effects of sex hormone since liver metabolism and inflammatory responses are highly influenced by estrogen levels and activity of estrogen receptor^67,68^. To induce acute liver failure, the median lobe and lateral lobe of the livers in 8-week-old male BALB/c mice were resected. Then, each MBL was transplanted immediately and harvested on day 5. To establish chronic liver failure model, 200 mg/kg of TAA (Sigma-Aldrich) was intraperitoneally injected 3 times a week in 6-week-old male BALB/c mice. After 6 weeks of TAA induction, MBLs were transplanted in interlobular space. After 2 weeks, host livers and transplanted MBLs were harvested and analyzed. Serum samples collected from the mice were subjected to cytometric bead array for analyzing systemic inflammation and serum chemistry for analyzing liver panels.

### Statistics and reproducibility

The sample size *n* represents the number of biologically independent replicates. All values were presented as mean±standard deviation (SD). Statistical analyses were performed using these independent values and graphs were generated using GraphPad Prism version 9.0. Statistical significance was determined by unpaired two-tailed *t-test* or one-way ANOVA with Tukey post-hoc test for multiple comparisons, otherwise stated separately. A value of *p* < 0.05 was considered significant (^*^p < 0.05; ^**^p < 0.01; ^***^p < 0.001). The exact *p* values for each figure were provided in Source Data. All microscopic images were representative, obtained from three independent experiments.

### Reporting summary

Further information on research design is available in the Nature Portfolio Reporting Summary linked to this article.

## Supplementary information

Supplementary Information

Description of additional Supplementary File

Supplementary Dataset 1

Reporting Summary

## Data Availability

All relevant data supporting the key findings of this study are available within the article and its Supplementary Information files or from the corresponding author upon reasonable request. All generated data in this paper are provided as Source data. Source data are provided with this paper.

## Source data

Source Data
