# Supplementary material for: Bioengineered liver crosslinked with nano-graphene oxide enables efficient liver regeneration via MMP suppression and immunomodulation
Source: Nat Commun. 2023 Feb 13;14:801. doi: 10.1038/s41467-023-35941-2 (PMC9925774; doi:10.1038/s41467-023-35941-2)
Supplement: Supplementary file 1 — Supplementary Information [file 41467_2023_35941_MOESM1_ESM.docx]

**Supplementary Information**

**Bioengineered liver crosslinked with nano-graphene oxide enables efficient liver regeneration via MMP suppression and immunomodulation**

Da-Hyun Kim^1^, Min-Ji Kim^1^, Seon-Yeong Kwak^2,3^, Jaemin Jeong^4^, Dongho Choi^4^, Soon Won Choi^1,5^, Jaechul Ryu^1,5^ and Kyung-Sun Kang^1,3*^

^1^ Adult Stem Cell Research Center and Research Institute for Veterinary Medicine, College of Veterinary Medicine, Seoul National University, Seoul, 08826, Republic of Korea,

^2^ Department of Agriculture, Forestry and Life Science, College of Agriculture and Life Science, Seoul National University, Seoul, 08826, Republic of Korea

^3^ Bio-MAX Institute, Seoul National University, Seoul, 08826, Republic of Korea

^4^ Department of Surgery, Hanyang University College of Medicine, Seoul, 04763, Republic of Korea

^5^ Institute of Bio & Nano Convergence, Biogo Co., LTD, Seoul, 08826, Republic of Korea

^*^Correspondence should be addressed to K.-S.K. (kangpub@snu.ac.kr)


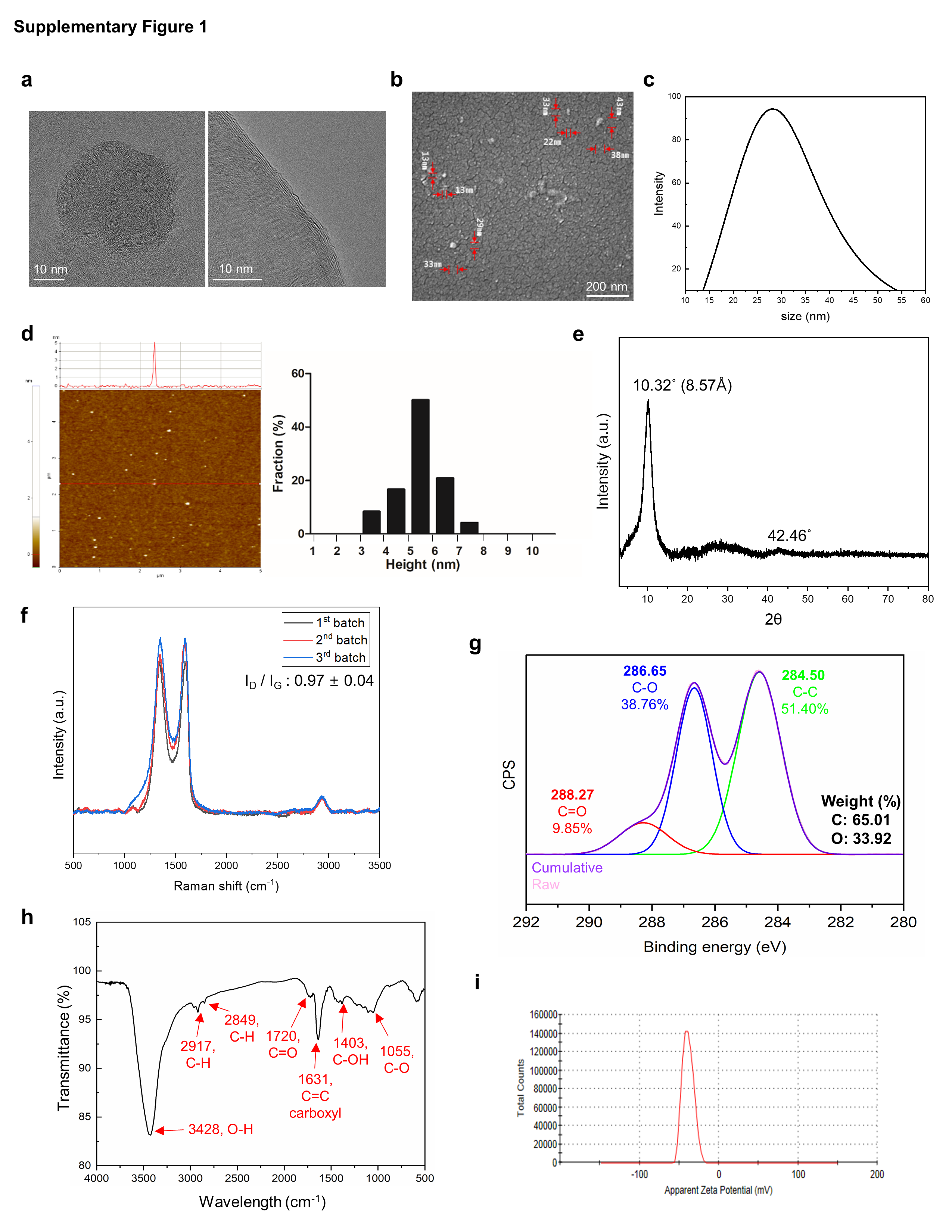


**Supplementary Fig. 1. Characterization of NGOs**

**a** High resolution transmission electron microscopy (HRTEM) images of NGOs showing spherical sheet-like morphology and lattice fringes (5 layers). **b** Scanning electron microscopy (SEM) images showing the lateral dimensions of NGO particles. **c** Size distribution curve of nano-graphene oxide (NGO) obtained by using a CPS instrument. The number of analyzed NGO particles was 389156.9 x 10^6^. **d** Atomic force microscopy images and height distribution of NGOs. (n=24) **e** X-ray diffraction analysis of NGOs demonstrating the distance between graphene layers (8.57Å). **f** Raman spectrum of NGOs showing D band and G band at 1350 cm^-1^ and 1580 cm^-1^, respectively. I_D_/I_G_ ratio of each spectrum was presented as a mean ± SD. (n=3) **g** The C1s spectrum of NGOs obtained by high resolution X-ray photoelectron spectroscopy, and the total content (%) of C and O was calculated. **h** Fourier transform infrared spectroscopy (FTIR) transmittance spectrum of NGOs showing the peaks of oxygen-containing functional groups at 3428, 2917, 2849, 1720, 1631, 1403 and 1055 cm^-1^. **i** Zeta potential of NGOs indicating moderate dispersion stability. Source data are provided as a Source Data file.


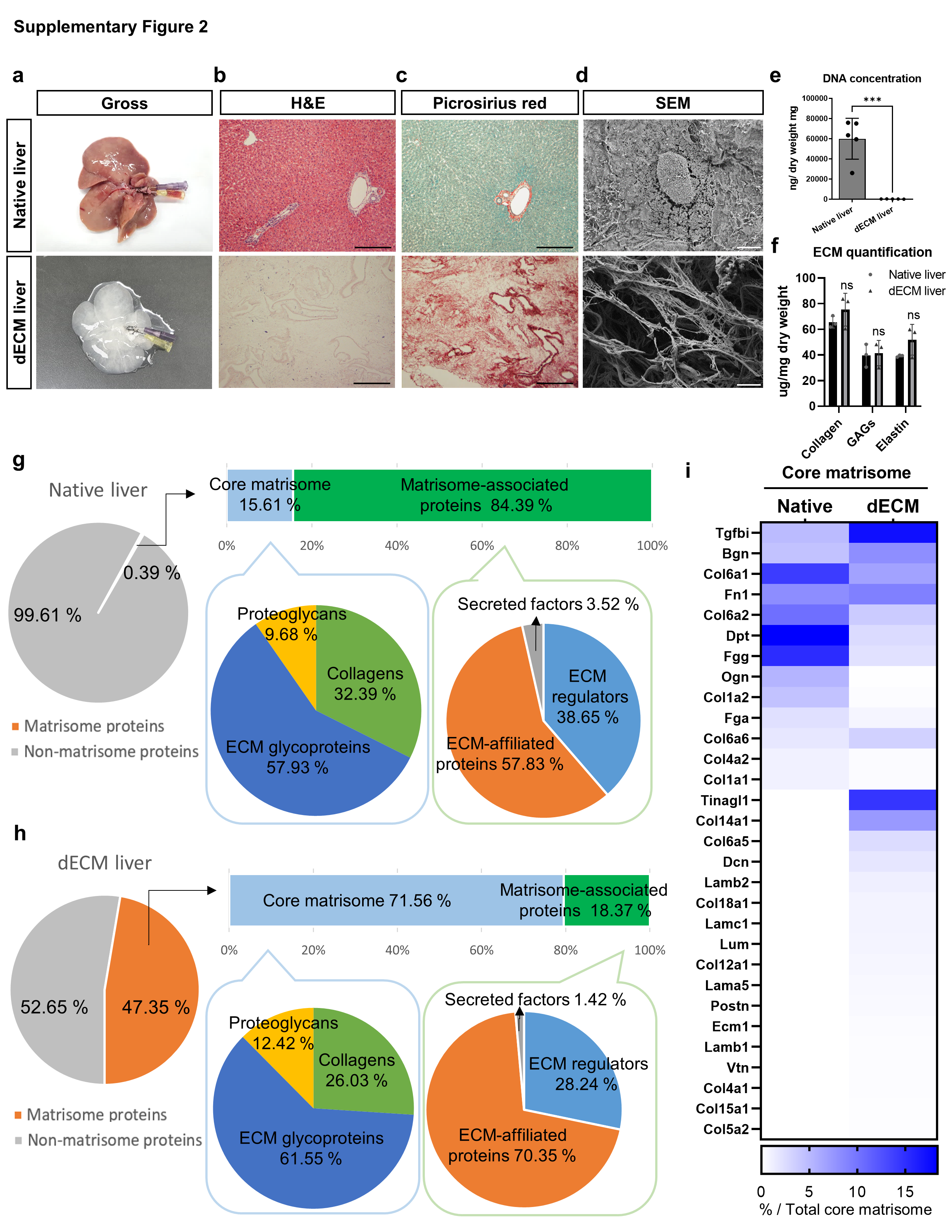


**Supplementary Fig. 2. Characterization of dECM liver scaffolds**

**a** Gross image of native liver and decellularized extracellular matrix (dECM) liver scaffold. **b-c** Histological examination of native liver and dECM liver by H&E staining (**b)** and picrosirius red staining **(c)**. Scale bar, 200 µm. **d** SEM analysis of native liver and dECM liver. Scale bar, 4 µm. **e** DNA quantification of native liver and dECM liver. (n=4) **f** Quantification of ECM contents, including collagen, glycosaminoglycans (GAGs) and elastin, in native liver and dECM liver. (n=4) **g-h** Profiles of matrisome proteins identified by mass spectrometry in native liver **(g)** and dECM liver **(h)**. **i** Heatmap demonstrating the proportion of top 30 core matrisome proteins to total core matrisomes in dECM liver. Quantitative data were presented as a mean ± SD. Statistical differences were determined by two-sided, unpaired student’s *t* test (***p < 0.001 versus native liver, ns; not statistically significant). Source data are provided as a Source Data file.


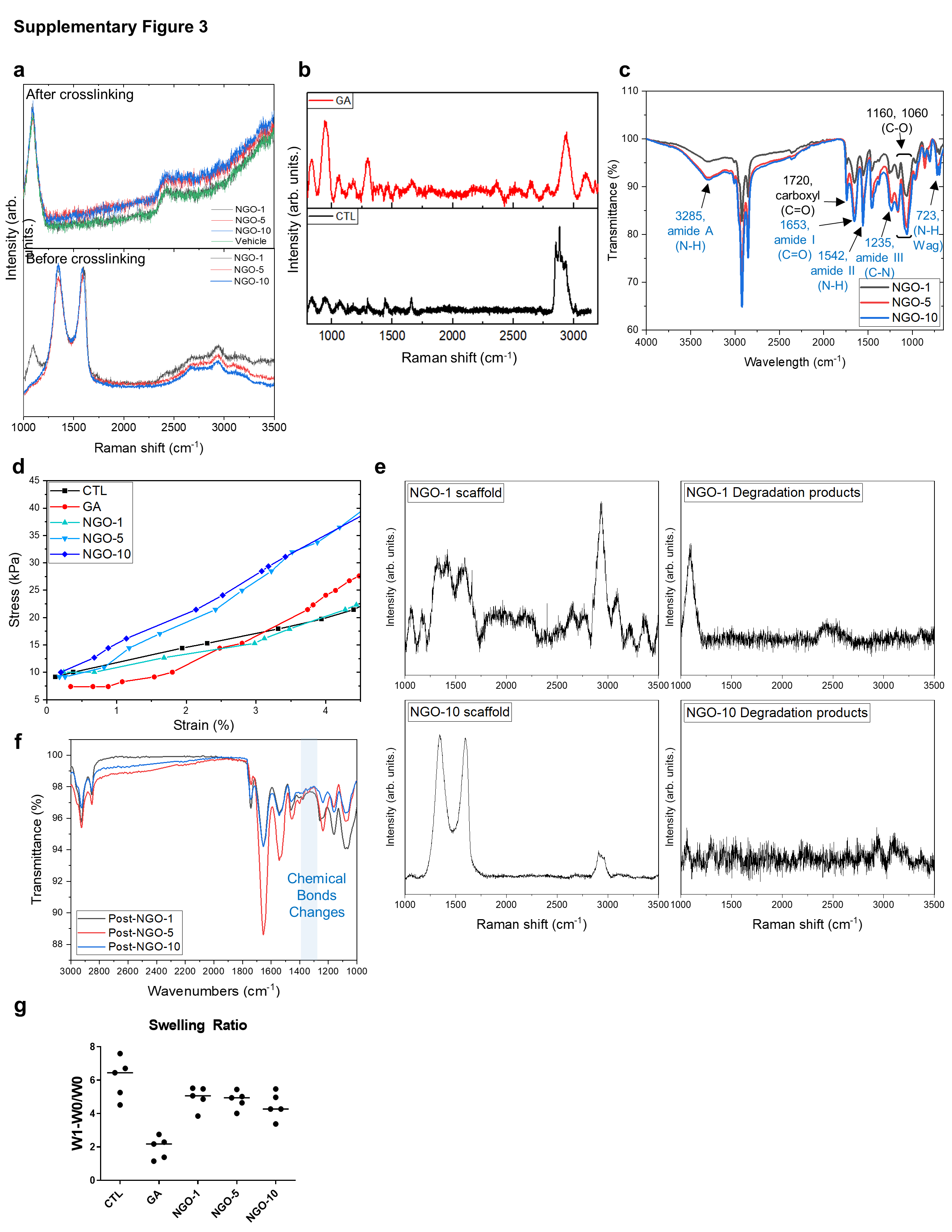


**Supplementary Fig. 3. Analysis of the chemical and physical properties of crosslinked scaffolds**

**a** Raman spectra of the perfusate before (bottom) and after (top) crosslinking of decellularized extracellular matrix (dECM) scaffolds with NGO-1, NGO-5 and NGO-10. **b** Raman spectra of the scaffolds without crosslinking (CTL) and crosslinked with glutaraldehyde (GA). **c** Fourier transform infrared spectroscopy (FTIR) spectra of the scaffolds crosslinked with different NGO concentrations: 1 μg/mL (NGO-1), 5 μg/mL (NGO-5) and 10 μg/mL (NGO-10). They showed similar patterns regardless of NGO concentration. **d** Representative stress-strain curve of the scaffolds in each group. R^2^ value of each group; CTL: 0.997, GA: 0.9819, NGO-1: 0.9794, NGO-5: 0.9937 and NGO-10: 0.9964. **e** Raman spectra of the scaffolds crosslinked with NGO-1 and NGO-10 after MMP-1 treatment (left) and their degradation products (right). **f** FTIR spectra of the scaffolds crosslinked with different NGO concentrations after MMP-1 treatment. **g** Swelling ratio of the crosslinked scaffolds in each group. (n=3) Source data are provided as a Source Data file.


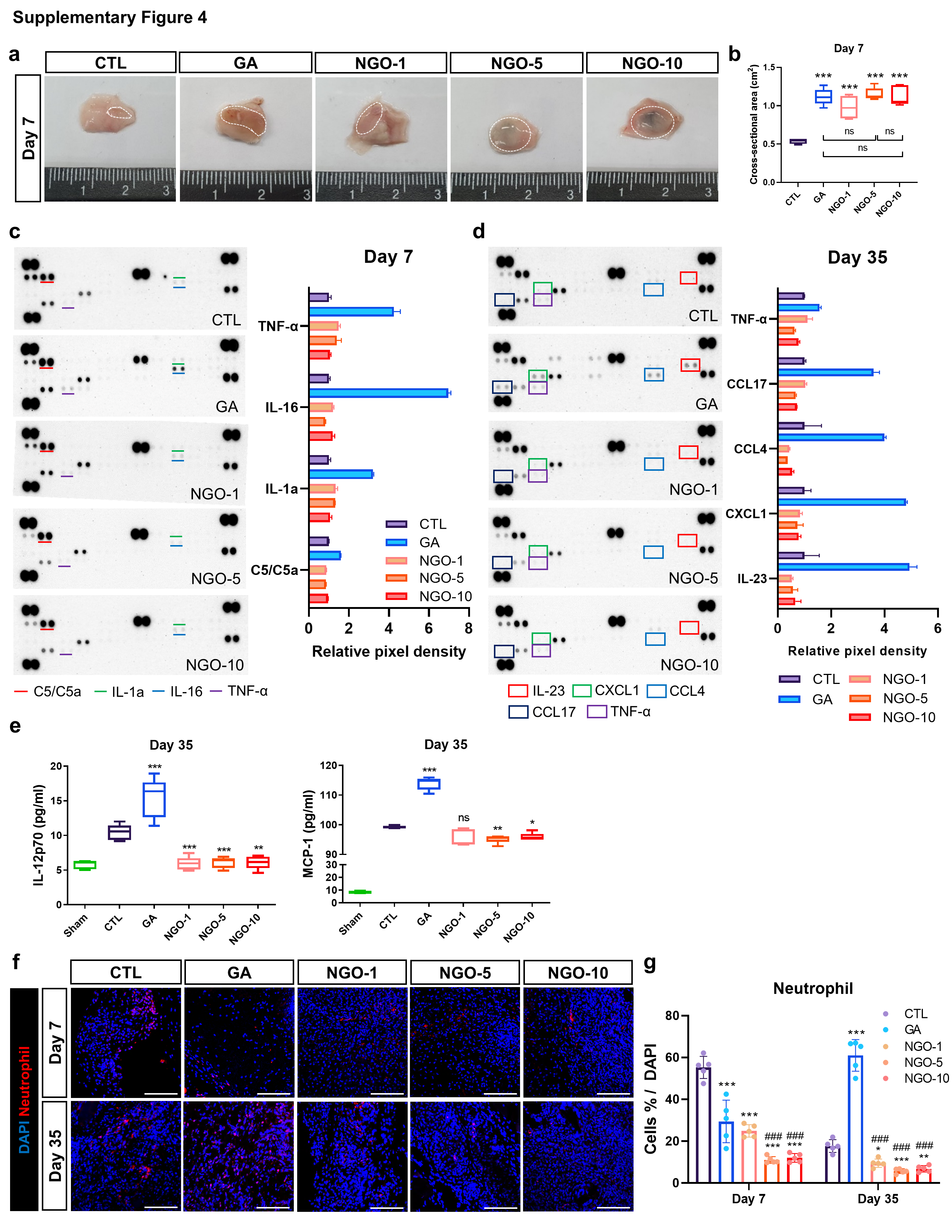


**Supplementary Fig. 4. Evaluation of immune responses after scaffold transplantation**

**a** The representative gross images of harvested scaffolds on day 7. **b** The box plot showing cross-sectional area of scaffold remnants after 7 days of transplantation. In each box plot, the center line within the box indicates the median, the edges of the box indicate the 25^th^ and 75^th^ percentile, and the whiskers indicate outliers outside the 10^th^ and 90^th^ percentile. (n=5) **c-d** Serum was collected from the mice receiving implantation of the scaffolds without crosslinking (CTL), crosslinked with gluataraldehyde (GA), 1 μg/mL NGO (NGO-1), 5 μg/mL NGO (NGO-5) and 10 μg/mL NGO (NGO-10). Global cytokine analysis of the serum collected from the mice on day 7 **(c)** and day 35 **(d)** and quantification of pixel density of each spot. (n=2) **e** Quantification of IL-12p70 and MCP-1 in the serum in each group on day 35 using CBA. (n=5) **f** Representative confocal images of implanted scaffolds on day 7 and day 35 probed with neutrophil antibody (red) and DAPI (blue). Scale bar, 100 µm. **g** Quantification of infiltrating neutrophils within the implanted scaffolds in each group. (n=5) Quantitative data were presented as a mean ± SD. Statistical differences between the groups were determined by ordinary one-way ANOVA with post-hoc Tukey test (*p < 0.05, **p < 0.01, ***p < 0.001 versus CTL. ###p < 0.001 versus GA, ns; not statistically significant). Source data are provided as a Source Data file.


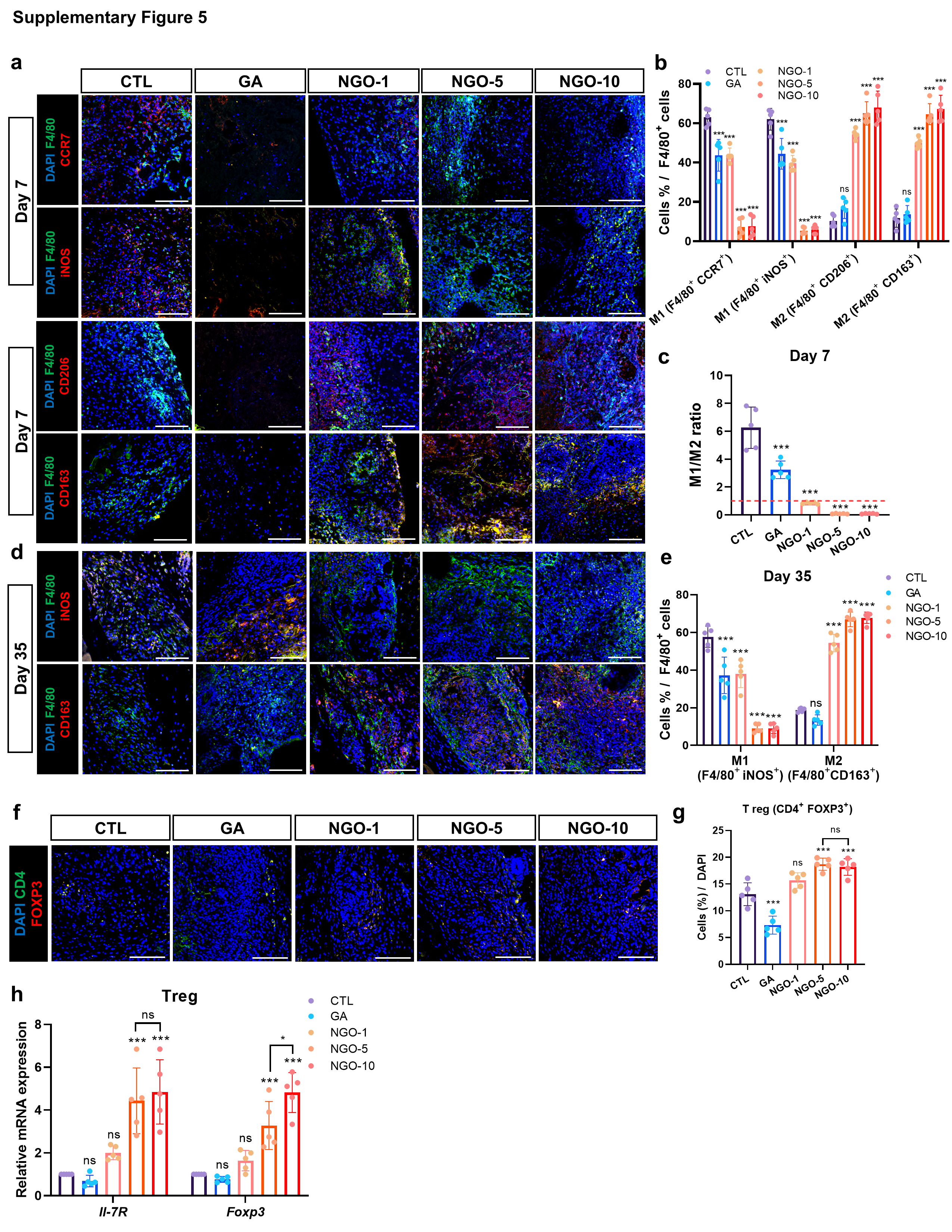


**Supplementary Fig. 5. Innate and adaptive immune responses within the transplanted scaffolds**

**a** Representative immunohistochemical images of the scaffolds on day 7 stained with respective M1 (CCR7 or iNOS; red) and M2 markers (CD206 or CD163; red), pan-macrophage marker (F4/80; green) and nuclei (blue). Scale bar, 100 µm. **b** Quantification of M1 or M2 macrophages normalized to F4/80^+^ cells in the implants on day 7. (n=5) **c** Calculation of M1/M2 ratio. (n=5) **d** Representative immunohistochemical images of the scaffolds on day 35 stained with M1 (iNOS; red) or M2 (CD163; red) marker, pan-macrophage marker (F4/80; green) and nuclei (blue). Scale bar, 100 µm. **e** Quantification of M1 or M2 macrophages normalized to F4/80^+^ cells in the implants on day 35. (n=5) **f** Representative confocal images of transplanted scaffolds harvested on day 35. CD4 (green), FOXP3 (red), DAPI (blue). Scale bar, 100 µm. **g** Quantification of CD4^+^ FOXP3^+^ Treg cells in each transplanted scaffold. (n=5) **h** qRT-PCR analysis of genes related to Treg cells, *Il-7R* and *Foxp3*, in each transplanted scaffold on day 35. (n=5) Quantitative data were presented as a mean ± SD. Statistical differences between the groups were determined by ordinary one-way ANOVA with post-hoc Tukey test (*p < 0.05, ***p < 0.001 versus CTL, ns; not statistically significant). Source data are provided as a Source Data file.


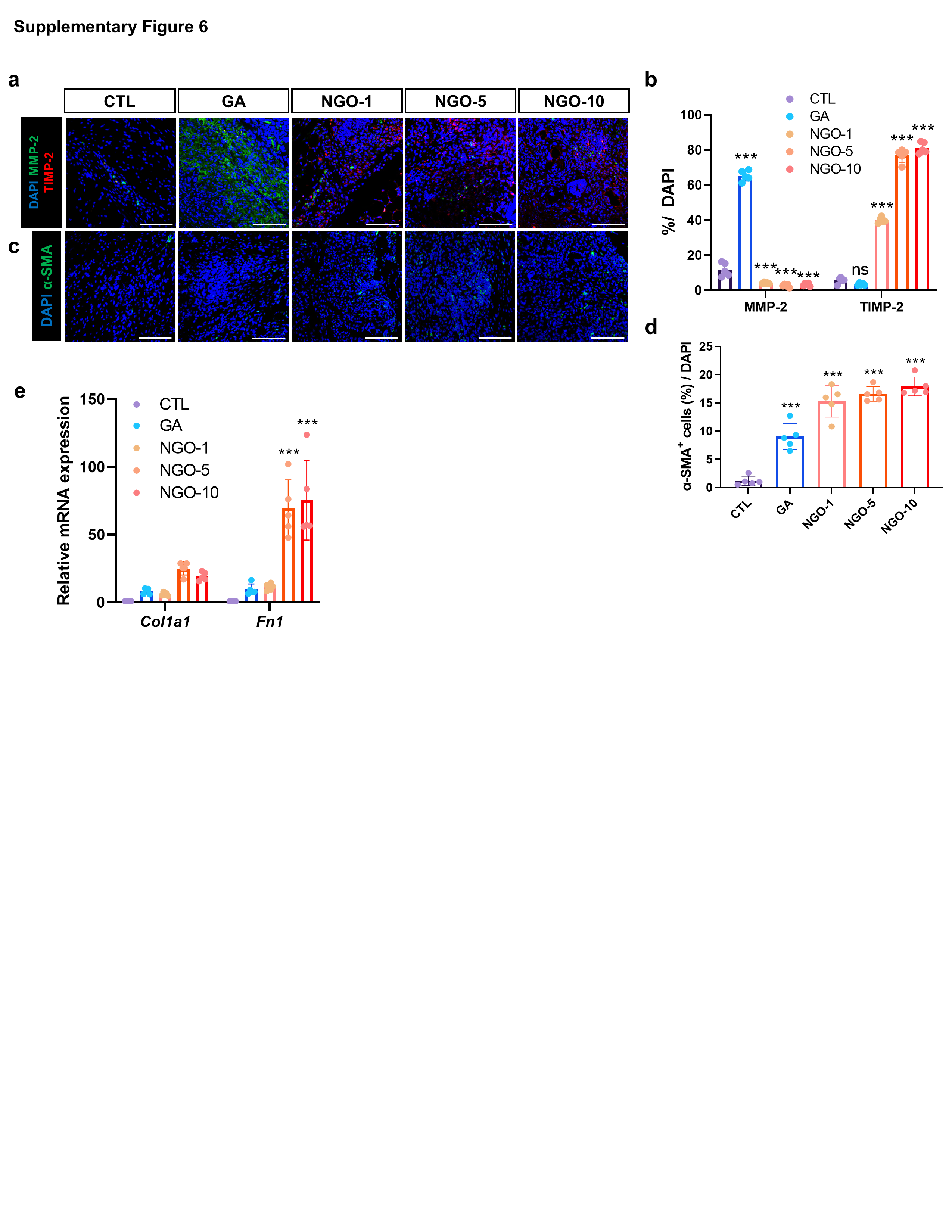


**Supplementary Fig. 6. Analysis of ECM degrading or protecting factors within the transplanted scaffolds**

**a-b** Representative confocal images **(a)** of the implanted scaffolds on day 35 and quantification **(b)** of cells expressing MMP-2 (green) and TIMP-2 (red). DAPI (blue). Scale bar, 100 µm. (n=5) **c-d** Representative confocal images **(c)** of the implanted scaffolds on day 35 and quantification **(d)** of cells expressing α-SMA (green). DAPI (blue). Scale bar, 100 µm. (n=5) **e** qRT-PCR analysis of ECM-related genes in each group on day 35. (n=5) Quantitative data were presented as a mean ± SD. Statistical differences between the groups were determined by ordinary one-way ANOVA with post-hoc Tukey test (***p < 0.001 versus CTL, ns; not statistically significant). Source data are provided as a Source Data file.


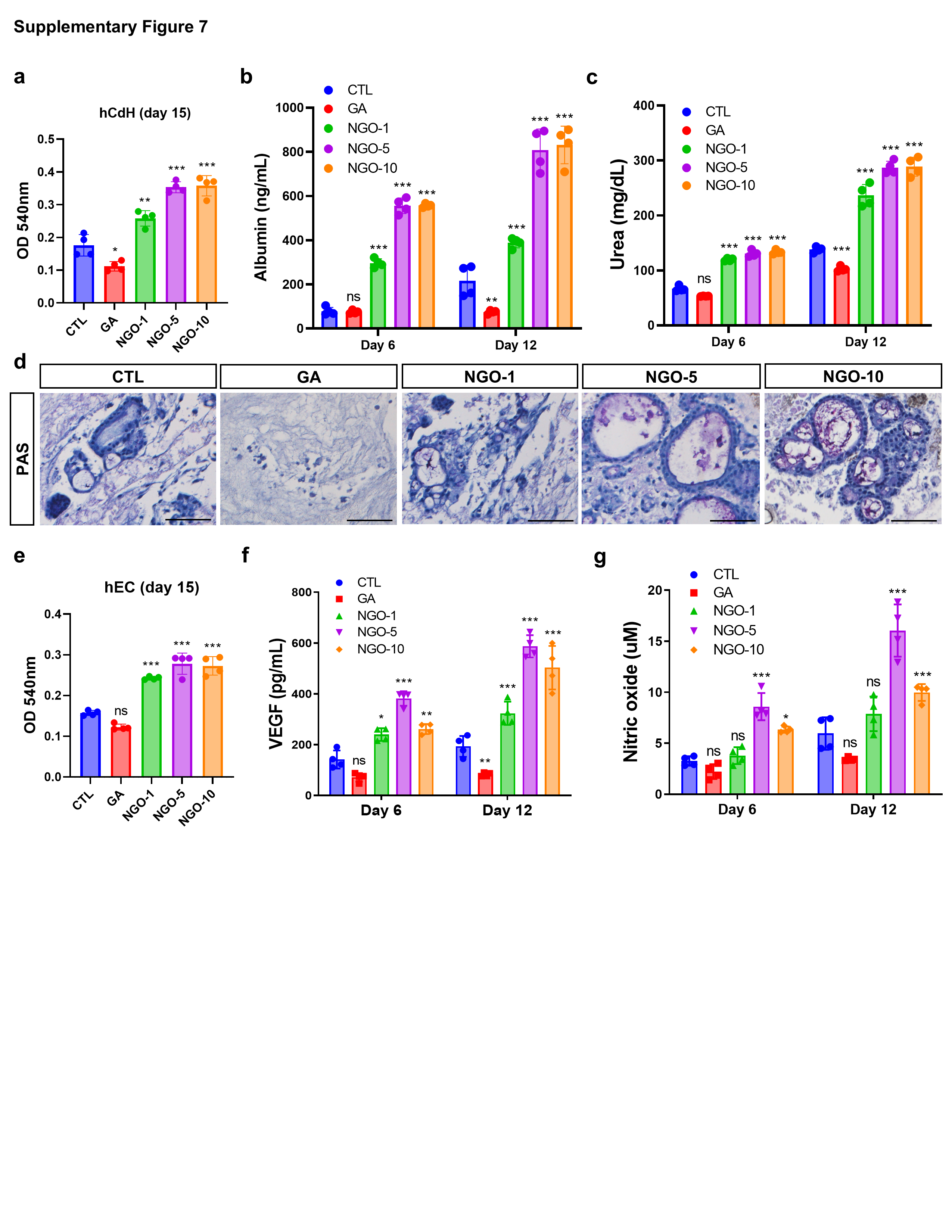


**Supplementary Fig. 7. Biocompatibility and functionality of human cell-seeded scaffolds**

**a** Quantification of viable human chemically derived hepatocytes (hCdHs) within each scaffold on day 15 by using MTT assay. (n=4) **b-c** The amount of secreted human albumin **(b)** and urea **(c)** from each scaffold seeded with hCdHs were quantified on day 6 and 12. (n=4) **d** Periodic acid-Schiff **(**PAS) staining of hCdH-seeded scaffolds for assessing glycogen synthesis. Scale bar, 400 µm. **e** MTT assay for evaluating the viability of endothelial cell (EC)-seeded scaffolds in each group. (n=4) **f-g** Secretion of VEGF **(f)** and nitric oxide **(g)** from each scaffold seeded with ECs were analyzed at the indicated time points. (n=4) Quantitative data were presented as a mean ± SD. Statistical differences between the groups were determined by ordinary one-way ANOVA with post-hoc Tukey test (**a, e**) and two-way ANOVA with post-hoc Tukey test (**b-c, f-g**) (*p < 0.05, **p < 0.01, ***p < 0.001 versus CTL, ns; not statistically significant). Source data are provided as a Source Data file.


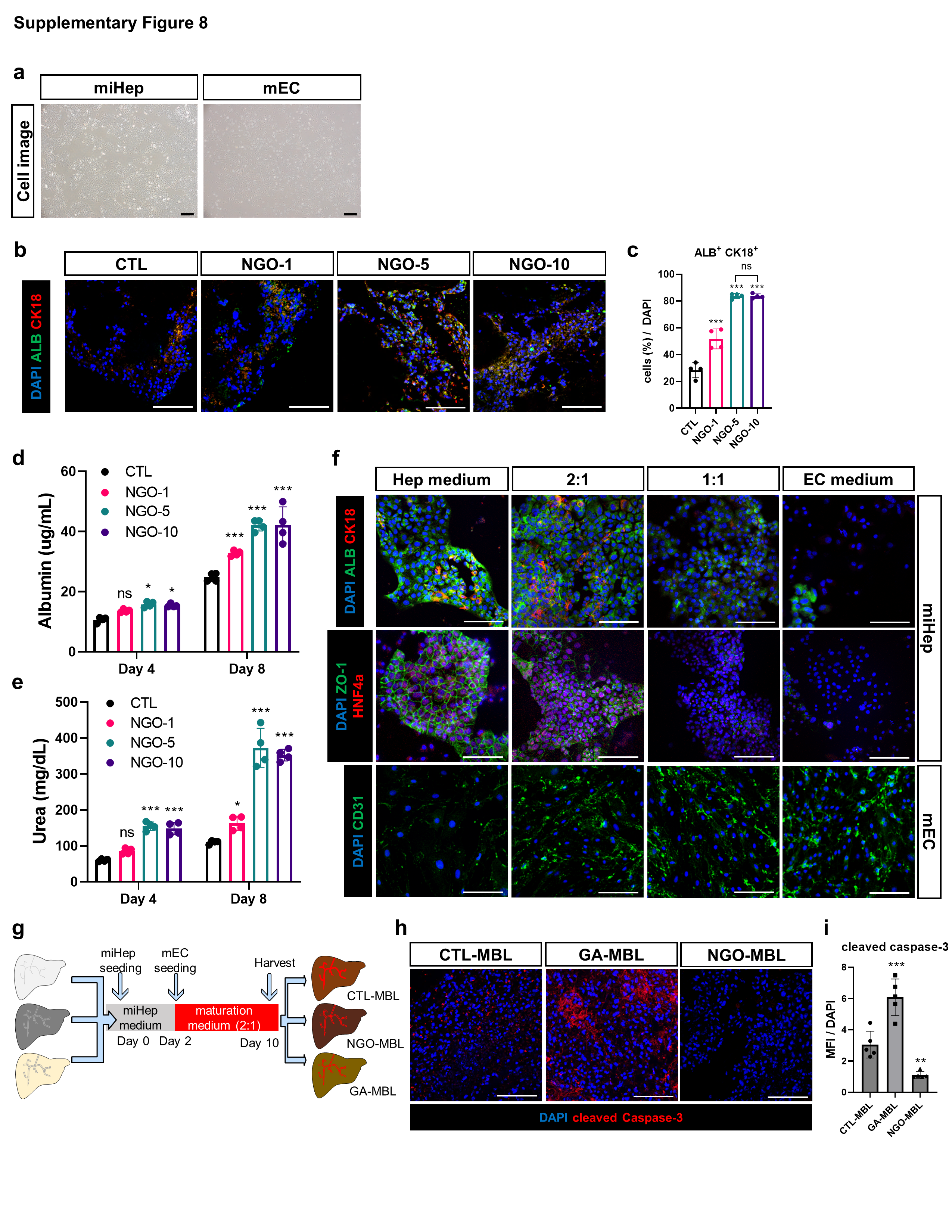


**Supplementary Fig. 8. The strategy for MBL reconstruction and its characterization**

**a** Bright field images of mouse induced hepatocytes (miHeps) and mouse endothelial cells (mECs). Scale bar, 200 µm. **b** Immunostaining of miHep-seeded scaffolds in each group. ALB (green), CK18 (red), DAPI (blue). Scale bar, 200 µm. **c** Quantification of ALB^+^ CK18^+^ cells in each group. (n=4) **d-e** Quantification of secreted albumin **(d)** and urea **(e)** from miHep-seeded scaffolds. (n=4) **f** Co-culture of miHeps and mECs maintained in miHep medium, a mixture of miHep medium and mEC medium in the ratio of 2:1 and 1:1, and EC medium respectively. The miHeps were stained with ALB (green) and CK18 (red), ZO-1 (green) and HNF4α (red). The mECs were stained with CD31 (green). The nuclei were stained with DAPI. Scale bar, 200 µm. Note that the optimal culture condition for both cells was 2:1. **g** Schematic illustration of mouse bioengineered liver (MBL) reconstruction using the scaffolds without crosslinking (CTL-MBL), crosslinked with nano-graphene oxide (NGO-MBL) and glutaraldehyde (GA-MBL). **h** Representative confocal images of CTL-MBLs, NGO-MBLs and GA-MBLs probed with cleaved Caspase-3 (red) and DAPI (blue). **i** Quantification of the cells expressing cleaved Caspase-3. Scale bar, 200 µm. (n=4) Quantitative data were presented as a mean ± SD. Statistical differences between the groups were determined by ordinary one-way ANOVA with post-hoc Tukey test (**c, i**) and two-way ANOVA with post-hoc Tukey test (**d-e**) (*p < 0.05, **p < 0.01, ***p < 0.001 versus CTL, ns; not statistically significant). Source data are provided as a Source Data file.


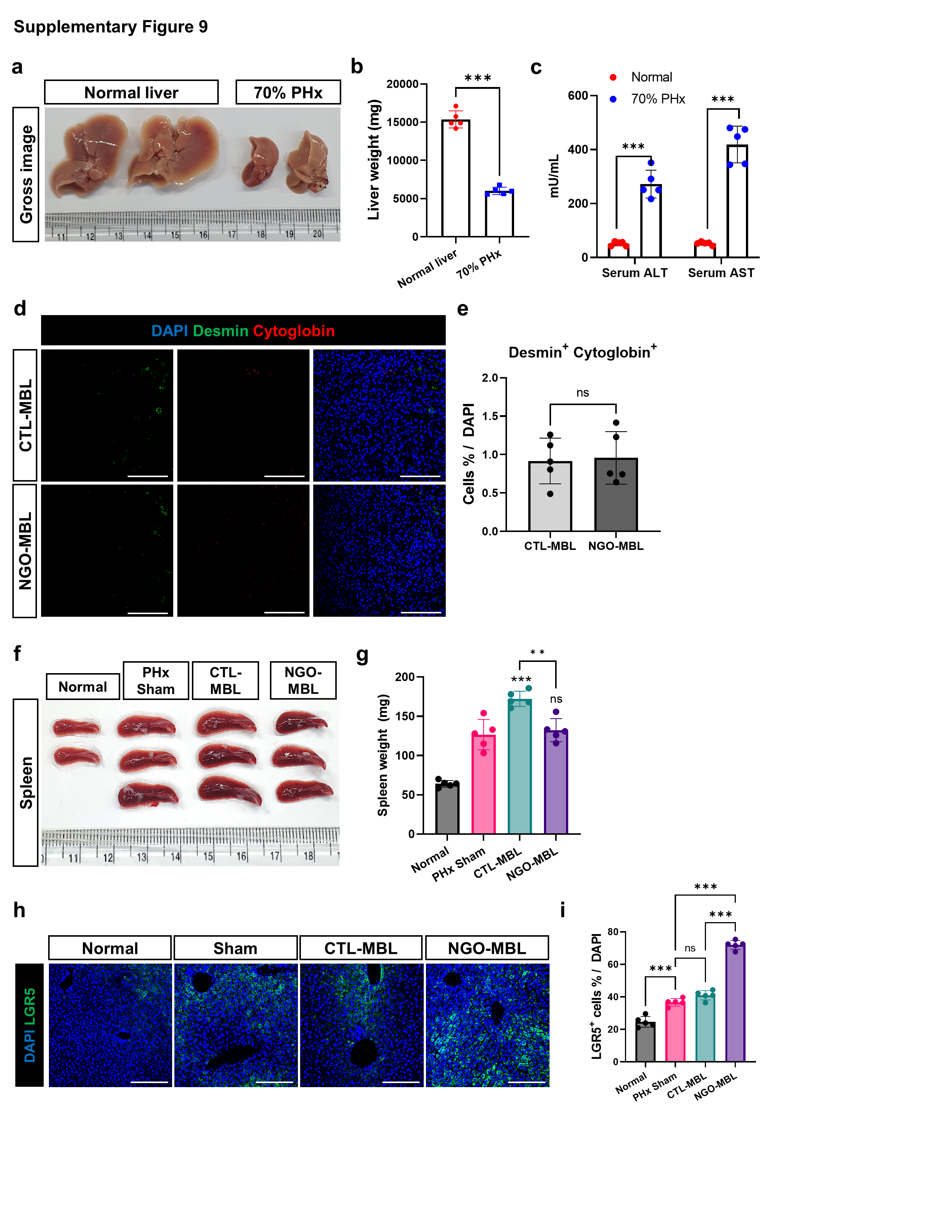


**Supplementary Fig. 9. Transplantation of MBLs into acute liver failure model**

**a** The gross images of normal livers and livers receiving 70% partial hepatectomy (70% PHx) to induce acute liver failure. **b** Quantification of liver weight in normal livers and 70% PHx livers. (n=5) **c** Serum levels of ALT and AST in normal group and 70% PHx group. (n=5) **d-e** Immunostaining **(d)** and quantification **(e)** of activated hepatic stellate cells (HSCs) in each mouse bioengineered liver (MBL) transplanted into acute liver failure model. Desmin (green), Cytoglobin (red), DAPI (blue). Scale bar, 100 µm. (n=5) **f** The representative images of spleen harvested from each group (Normal; the mice with normal liver, PHx sham; the mice receiving only 70% PHx, CTL-MBL or NGO-MBL; the mice receiving both 70% PHx and transplantation of CTL-MBL or NGO-MBL). **g** Quantification of spleen weight in each group. (n=5) **h** Immunostaining of transplanted CTL-MBLs and NGO-MBLs. LGR5 (green), DAPI (blue). Scale bar, 100 µm. **i** Quantification of LGR5^+^ cells in the liver sections of each group. (n=5) Quantitative data were presented as a mean ± SD. Statistical differences between the groups were determined by unpaired, two-tailed student’s *t* test (**b-c, e**) and ordinary one-way ANOVA with post-hoc Tukey test (**g, i**) (**p < 0.01, ***p < 0.001 versus CTL, ns; not statistically significant). Source data are provided as a Source Data file.


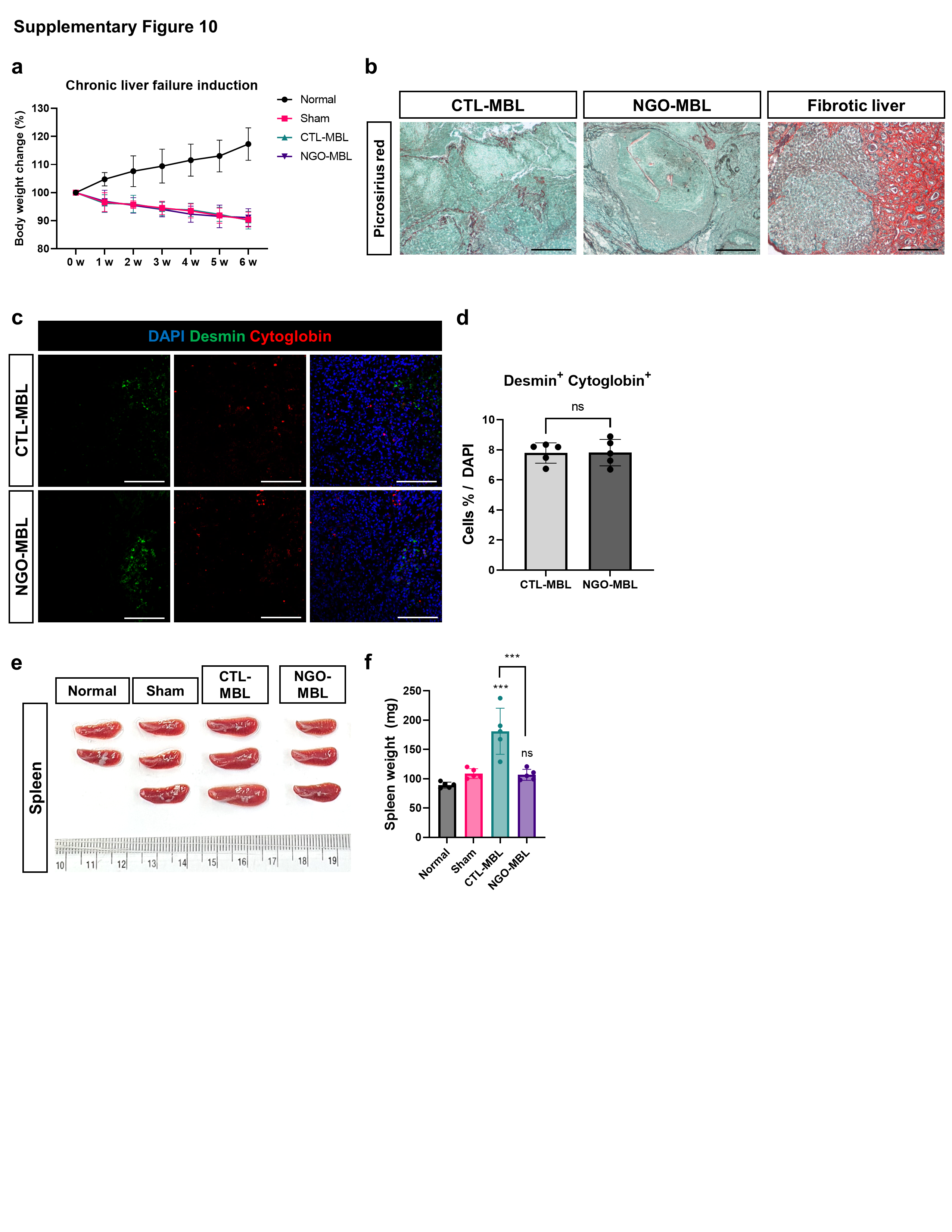


**Supplementary Fig. 10. Transplantation of MBLs into chronic liver failure model**

**a** Body weight change of the mice in each group during thioacetamide (TAA) induction. (n=5) **b** Picrosirius red staining of mouse bioengineered livers (MBLs) after transplantation into the chronic liver failure mice and fibrotic rat liver. MBLs were fabricated by using the scaffolds without crosslinking (CTL-MBLs) and the scaffolds crosslinked with nano-graphene oxide (NGO-MBLs). Scale bar, 200 µm. **c-d** Immunostaining **(c)** and quantification **(d)** of activated hepatic stellate cells (HSCs) in each MBL transplanted into chronic liver failure model. (Green: Desmin, Red: Cytoglobin, Blue: DAPI). Scale bar, 100 µm. (n=5) **e** Representative harvested spleen images of normal mice (Normal), mice induced with TAA (Sham), mice transplanted with CTL-MBLs (CTL-MBL) and NGO-MBLs (NGO-MBL) after TAA induction. **f** Quantification of spleen weight in each group. (n=5) Quantitative data were presented as a mean ± SD. Statistical differences between the groups were determined by unpaired, two-tailed student’s *t* test (**d**) and ordinary one-way ANOVA with post-hoc Tukey test (**f**) (***p < 0.001, ns; not statistically significant). Source data are provided as a Source Data file.

**Supplementary Table. 1. List of primers used for qRT-PCR.**

All sequences are mouse-specific.

| **Genes** | **5’-Forward sequence-3’** | **5’-Reverse sequence-3’** |
| --- | --- | --- |
| *Gapdh* | GCCCAGAACATCATCCCTG | GACGGACACATTGGGGGTAG |
| *Cd86* | AGCACGGACTTGAACAACCA | TGTAAATGGGCACGGCAGAT |
| *iNos* | GGTGAAGGGACTGAGCTGTT | TGAAGAGAAACTTCCAGGGGC |
| *Il-1β* | TGCCACCTTTTGACAGTGATG | TGATACTGCCTGCCTGAAGC |
| *Il-12* | ATGTGTCTCCCAAGGTCAGC | CTTCAGCAGGTTTCGGGACT |
| *Cd163* | AGACACACGGAGCCATCAAA | GGAGCGTTAGTGACAGCAGA |
| *Cd206* | GTGGAGTGATGGAACCCCAG | CTGTCCGCCCAGTATCCATC |
| *Arg1* | GCAGTGGCTTTAACCTTGGC | CTGTGATGCCCCAGATGGTT |
| *Il-10* | TGCTGCCTGCTCTTACTGAC | CTAGGAGCATGTGGCTCTGG |
| *Tlr1* | AGCGGTTCTCGGGTACAAAG | GCTGACGGACACATCCAGAA |
| *Tgfb1* | AGCTGCGCTTGCAGAGATT | AGCCCTGTATTCCGTCTCC |
| *Mmp1* | ACTACAACTGACAACCCAAGAAAG | CCTCTCTTGAAAGGAGATGCCT |
| *Mmp2* | TCAACGGTCGGGAATACAGC | GTAAACAAGGCTTCATGGGGG |
| *Mmp9* | AAAGGCAGCGTTAGCCAGA | GAAGACCACAAAAGTCGGCTG |
| *Timp1* | GAAGCCTGGAGGCAGTGATT | AGGGGGCCATCATGGTATCT |
| *Timp2* | CAGCCTCTCCCGTCTTTTGT | GTGGCTAGAAACCCCAGCAT |
| *Col1a1* | CTACTACCGGGCCGATGATG | AGTGGCACATCTTGAGGTCG |
| *Fn1* | CGAGGTGACAGAGACCACAA | CTGGAGTCAAGCCAGACACA |
| *Il-7R* | TCTGGAGAAAGTGGAAATGCCC | CTGTGGCACCAGAAGGAGTG |
| *Foxp3* | TGCAGTTCCTTTGTGTCCG | ATAGTCACCCCAACACAGC |
| *α-Sma* | GTACCACCATGTACCCAGGC | GCTGGAAGGTAGACAGCGAA |
| *Vimentin* | TCCAGAGAGAGGAAGCCGAA | TTCAAGGTCAAGACGTGCCA |
| *Afp* | AAACTCTGGCGATGGGTGTT | CTGGAAACTGGAAGGGTGGG |
| *Dlk1* | AAGAGTACCCCTCTCCTCACC | GCGAAGCATGTGGTTGTAGC |
| *Ptn* | GAGTGAAGGCAGGATCAGGTT | TTTTCTTGTGCTCTGGGGCTT |

**Supplementary Methods**

**Histological examination**

Tissue samples were fixated in 4% formaldehyde overnight at 4℃ and subjected to tissue processing. The dehydrated samples were embedded in paraffin and cut into 10 μm sections. Tissue sections were deparaffinized and hydrated with a series of ethanol washes with decreasing concentration. Tissue slides were subjected to picrosirius red staining (0.1% Direct red 80; Sigma, 0.1% Fast green FCF; Sigma) or hematoxylin (Sigma) & eosin (Muto Pure Chemicals, Japan) staining or PAS staining (ab150680, Abcam, England) according to the manufacturer’s instructions. After staining, slides were then washed with tap water and dehydrated with an ascending series of ethanol ranging from 70% to 100% followed by xylene washes. Samples were visualized with Nikon light microscope and NIS-Elements software (Nikon, Japan).

**SEM**

Specimens were fixated with 4% glutaraldehyde and 1% paraformaldehyde in a buffered solution of 0.1 M cacodylate buffer. Then, the samples were rinsed with cacodylated buffer and dehydrated through a series of graded ethanol. After critical point drying, sputter coated samples were visualized with Variable Pressure Field Emission Scanning Electron Microscope (SUPRA55VP, Carl Zeiss, Germany).

**Quantification of ECM components**

For comparison of ECM contents in native livers and dECM livers, Sircol soluble collagen assay, Fastin Elastin assay, Blyscan sulfated Glycosaminoglycan assay were used. (All purchased from Biocolor, UK). Insoluble collagen of the crosslinked scaffolds after exposure to MMPs were extracted using Sircol insoluble collagen assay (Biocolor). The quantified results were normalized to the dry weight of each sample (Supplementary Gig. 3g). For this, swelling ratio of each group was measured for normalization as described in “Fabrication of decellularized liver scaffolds and characterization”.

**Immunofluorescence staining**

Prior to staining, formalin fixed, paraffin-embedded tissue sections were deparaffinized with xylene and subsequently hydrated through a series of decreasing ethanol concentration. Cells or tissue sections were fixated with 4% formaldehyde in PBS for 10 minutes, followed by antigen retrieval with sodium citrate buffer (pH 6.0, Sigma). After blocked with 5% normal goat serum (Vector Laboratories, Switzerland) in 0.1% Triton X-100 (Sigma Aldrich) for an hour at room temperature, the samples were probed with the following primary antibodies overnight at 4℃: anti-Collagen type I (GTX26308, GeneTex, USA, 1:100), anti-Collagen type IV (ab19808, Abcam, 1:100), anti-neutrophil (ab2557, Abcam, 1:100), anti-F4/80 (ab6640, Abcam, 1:100), anti-CCR7 (ab221209, Abcam, 1:100), anti-iNOS (ab15323, Abcam, 1:100), anti-CD206 (ab8918, ab64693, Abcam, 1:100), anti-CD163 (ab182422, Abcam, 1:100), anti-MMP-1 (MAB901, R&D systems, USA, 1:100), anti-MMP-2 (AB19015, Sigma-Aldrich, 1:100), anti-MMP-9 (AB19016, Sigma-Aldrich, 1:100), anti-TIMP-1 (sc-21734, Santa Cruz Biotechnology, USA, 1:50), anti-TIMP-2 (sc-5539, Santa Cruz Biotechnology, 1:50), anti-α-SMA (ab184675, Abcam, 1:100), anti-CD86 (ab239075, Abcam, 1:100), anti-CD68 (ab955, Abcam, 1:100), anti-CD4 (130-120-819, Miltenyi Biotec, Germany, 1:50), anti-FOXP3 (130-120-674, Miltenyi Biotec, 1:50), anti-ALB (GTX102419, Genetex, USA, 1:100), anti-CK18 (MAB3234, Merck Millipore, USA, 1:100), anti-ZO-1 (40-2200, Invitrogen, USA, 1:100), anti-HNF4a (MA1-199, Invitrogen, 1:100), anti-CD31 (ab28364, Abcam, 1:100), anti-cleaved Caspase3 (9664, Cell signaling technology, USA, 1:100), anti-Desmin (sc-23879, Santa Cruz Biotechnology, 1:50), and anti-Cytoglobin (GTX117571, GeneTex, 1:100). After washing with PBS containing 0.025% Triton X-100, the sections were incubated with following fluorescent-dye conjugated secondary antibodies for an hour at room temperature: Alexa Flour 488-labeled (A11001, A11006, A11008), 594-labeled (A11005, A11012), 647-labeled (A21235) and 488-labeled streptavidin (S11223), all purchased from Invitrogen. Sections were stained with DAPI (sc-3598, Santa Cruz Biotechnology) for nuclei detection, followed by mounting with fluorescent mounting medium (S302380, DAKO, Denmark). The images were visualized in the randomized fields by Eclipse TE 2000 confocal laser scanning microscope (Nikon, Japan) with EZ-C1 3.8 program. For measuring the degree of co-localization between biotinylated NGOs and ECM fibers (collagen I or collagen IV), the overlap coefficient was calculated by using BioImaging and Optics Platform (BIOP) ImageJ plugins which was developed by Ecole Polytechnique Fédérale de Lausanne (EPFL, Switzerland). F4/80^+^ CCR7^+^ cells or F4/80^+^ iNOS^+^ cells were regarded as mouse M1 macrophages and F4/80^+^ CD206^+^ cells or F4/80^+^ CD163^+^ cells were regarded as mouse M2 macrophages. The cells expressing respective markers were quantified by Image J software. For calculation of M1/M2 ratio, the number of M1 and M2 macrophages was normalized to F4/80^+^ cells, and then M1 (F4/80^+^ CCR7^+^ / F4/80^+^ or F4/80^+^ iNOS^+^/ F4/80^+^) was divided by M2 (F4/80^+^ CD206^+^ / F4/80^+^ or F4/80^+^ CD163^+^ / F4/80^+^).

**Young’s modulus**

Prior to experiments, the crosslinked scaffolds were prepared by air-drying for 24 hours and subjected to Young’s modulus measurement using Univert (CellScale, Canada). The elastic modulus of each crosslinked scaffold was obtained with a loading speed of 1 mm/min. The maximum load was measured by stretching the samples until the final fracture. During the test, the displacement (mm) – force curve (N) was automatically recorded and subsequently converted into a strain (%) – stress (kPa) curve considering the initial length of the sample.

**Ninhydrin assay**

To estimate the degree of crosslinking, free amino acid groups within the crosslinked scaffolds after exposure to MMP-1, MMP-2 and MMP-9 were respectively quantified by using ninhydrin reaction. Each scaffold was soaked into the mixture of 1 mL of ninhydrin reagent (N7285, Sigma) and 2 mL of distilled water. The samples were boiled at 100℃ for 10 minutes and cooled down to room temperature. After 3 mL of 95% ethanol was added to each tube, the absorbance at wavelength of 570 nm was measured using a microplate reader (Infinite M200 pro, Tecan, Switzerland).

**^1^H NMR and STD-NMR**

The interactions among NGOs and the catalytic domains of MMPs were investigated by using an 850 Hz NMR spectrometer (AVANCE Ⅲ HD, Bruker, Germany) at 37℃. All ^1^H and STD NMR spectra were measured at 850.22 Hz using a 5 mm TCI cryogenic probe. For NMR analysis, 100 μg/mL of MMP-1 (LS004217, Worthington, USA), MMP-2 (#420-02, Peprotech) and MMP-9 (17104019, Gibco), were activated by cleaving pro-peptides. After enzymatic activation, MMPs were incubated with 100 μg/mL of NGOs at 37℃. Then, NGO alone, MMP alone and MMP mixed with NGO formulated in H_2_O supplemented with 10% D_2_O were subjected to NMR analysis. 0.1M of L-glutamate (G1251, Sigma) and L-histidine (H8125, Sigma) were also subjected to ^1^H NMR analysis to examine the interactions with NGOs following the same procedures as described. For saturation transfer difference (STD)-NMR analysis, the spectra for both off-resonance (reference spectra) and on-resonance were collected. STD spectra were obtained by subtracting on-resonance from off-resonance spectra. The NMR spectra were produced using OriginPro 2022b program (USA).

**MMP activity assay**

For measuring the activity of MMP-1, collagenase activity assay kit (ab196999, Abcam) was used. Collagenase mixtures incubated with different concentrations (1 μg/mL, 5 μg/mL and 10 μg/mL) of NGOs for 10 minutes at 37℃ were reacted with collagen substrates. 1,10-Phenanthroline was used as a collagenase inhibitor control. According to the manufacturer’s instructions, the absorbance at a wavelength of 345 nm was measured in a kinetic mode using microplate reader (Infinite M200 pro, Tecan, Switzerland). The activity of collagenase was calculated by using the absorbance measured after 5 minutes and 15 minutes of enzymatic reaction. For measuring the activities of MMP-2 and MMP-9, activated MMP subtypes were incubated with different concentrations of NGOs for 15 minutes at 37℃. In this case, doxycycline was used as a MMP inhibitor control^1^. Subsequently, MMP substrates in MMP activity assay kit (ab112146, Abcam) were added into the mixture of each group. The fluorescence intensity at 490/525 nm (Ex/ Em) was measured 15 minutes after adding substrates using multiple plate reader (Victor 3, Perkin Elmer, USA). Furthermore, the catalytic activities of MMPs were also measured after MMPs were reacted with the dECM scaffolds crosslinked with different concentrations of NGOs (1 μg/mL, 5 μg/mL and 10 μg/mL). To demonstrate whether adsorbed serum proteins could influence the MMP inhibitory effects of NGOs, NGOs were incubated with 10% FBS for 20 minutes at 37℃ and then subjected to reaction with MMPs.

**Zinc ion chelating activity**

As previously reported, dithizone (#43820, Sigma) was employed to confirm the zinc chelating properties of NGOs^2^. 10 μM of zinc chloride (#208086, Sigma) in 15 mM HEPES buffer (pH 7.5) were incubated with vehicle (water), different concentrations of NGOs (1, 5 and 10 μg/mL) and 10 μM of EDTA (positive control) for 5 minutes. Then, 250 μM of dithizone or blank (DMSO) were added and the absorbance at a wavelength of 540 nm was measured using microplate reader. The absorbance measurements of Zn^2+^ concentration incubated with dithizone are normalized to those incubated with DMSO (vehicle).

**qRT-PCR**

Total RNA was extracted from the implants using NucleoZOL (Macherey-Nagel, Germany), 1 μg of RNA was used to synthesize cDNA using Superscript Ⅲ First-Strand Synthesis System (Invitrogen). Subsequently, cDNA was amplified by using ABI 7300 Real time PCR system (Applied Biosystems, USA) with SYBR Green PCR Master Mix (Applied Biosystems). Relative mRNA expression of target genes was quantified by 2^(ΔΔ threshold cycle)^ (2^−ΔΔCT^) method and normalized to GAPDH (housekeeping gene) expression. The mouse primer sequences used for qRT-PCR were provided in Supplementary Table 1.

**Global cytokine analysis**

The serum samples harvested on day 7, day 35 and day 60 from the mice receiving scaffold transplantation were analyzed by using Proteome Profiler Mouse Cytokine Array Kit Panel A (ARY006, R&D systems). Following the manufacturer’s protocols, 100 μL of each serum was incubated with the nitrocellulose membrane spotted with 40 different antibodies to mouse cytokines and chemokines at 4℃ overnight. After each membrane was incubated with Streptavidin-HRP at room temperature for 30 minutes, the membrane was detected by using ChemiDoc MP Imaging Systems (Bio-Rad, USA). The intensity of each spot was measured using ImageJ.

**Cytometric bead array**

Mouse inflammation kit (BD552364, BD bioscience, USA) was used to quantify the levels of pro-inflammatory cytokines in the serum samples. According to the manufacturer’s protocols, serum samples collected from mice transplanted with crosslinked scaffolds, CTL-MBLs or NGO-MBLs at the indicated time points were incubated with capture bead mixtures (mouse IL-12p70, MCP-1 and TNF-α) and PE detection reagents for 2 hours at room temperature. After washed with wash buffer, the samples were subjected to flow cytometry analysis. Acquisition strategy was established with cytometer setup beads according to the manufacturer’s protocol. Based on the template, the singlet bead populations were first gated using FSC-H/SSC-H dot plot, followed by gating each capture bead in FL2-H/ FL3-H dot plot (each capture bead can be distinguished by fluorescence intensity). After gating, corresponding MFI of FL2-H was quantified. MFI values of PE channel were analyzed using FlowJo software. By using MFI values, the concentration of each protein in the serum samples was calculated according to the standard curve.

**Flow cytometry**

To analyze whether the seeded CD14^+^ cells were polarized into either M1 or M2 like macrophages, the cells were extracted from the scaffolds by using the protocol modified from the report regarding the digestion of murine liver^5^. Briefly, the scaffolds were minced and incubated with 2500 U/mL of collagenase type Ⅳ (17104019, Gibco) and 1 mg/mL of DNase 1 (10104159001, Roche, Switzerland) for 20 minutes at 37℃ CO_2_ incubator. After filtering with 100 μm Nylon cell strainer (352360, Falcon, USA), the cells were washed with isolation buffer (4.8% bovine serum albumin and 2mM EDTA in Hank’s balanced salt solution) 3 times by centrifugation at 4℃. Then the cell pellets were suspended with PBS containing 2% FBS and subsequently incubated with the following antibodies (1:20 dilution) at 4℃ for 15 minutes; CD14 (555397) and CD86 (555659) for M1; CD14, CD163 (563887) and CD206 (555954) for M2. All antibodies were purchased from BD bioscience. The cells were washed with PBS and subjected to flow cytometry (FACS Calibur, BD bioscience). Data were acquired by BD CellQuest Pro (v.6.0, BD bioscience) and analyzed using FlowJo software (v.10.6.1). After FSC-A/SSC-A live cell gating, CD14-FITC was gated for distinguishing pan-macrophage populations. The boundary for negative and positive populations was determined by negative control populations. Among CD14+ populations, M1 populations were identified based on the expression of CD86 and CD163+ CD206+ (double positive) populations were regarded as M2 populations.

**MTT assay**

The metabolic activity of cells within the crosslinked scaffolds was measured using tetrazolium-based colorimetric assay. On day 15, cell-seeded scaffolds plated in 12-well plates were washed with PBS and subsequently incubated with 10% of MTT reagent (Sigma Aldrich) diluted in respective culture media for 4 hours at 37℃. After 4 hours, MTT reagent was removed and dimethyl sulfoxide was added. Then the plates were placed on the microplate shaker for thorough mixing until enough cell lysis. The absorbance of MTT formazan at wavelength of 570 nm was measured using a microplate reader.

**Enzyme-linked immunosorbent assay (ELISA)**

For assessment of biocompatibility of crosslinked liver scaffolds in each group, the conditioned media was harvested from hCdH-seeded scaffolds and EC-seeded scaffolds respectively. Secretion of albumin and urea from hCdH-seeded scaffolds were quantified by using Albumin ELISA kit (E80-129, Bethyl Laboratories, USA) and Quanti Chrome Urea Assay kit (DIUR-100, BioAssay Systems, USA). Secretion of human VEGF was analyzed with human VEGF Quantikine ELISA kit (DVE00, R&D systems). Nitric oxide production was quantified with NO Plus Detection Kit (21023, iNtRON, Republic of Korea). For demonstrating the effects of NGOs on miHep differentiation and the liver-specific functionality of MBLs, the culture supernatant of each sample was subjected to mouse Albumin ELISA kit (ab108792, Abcam) and Quanti Chrome Urea Assay kit. To quantify ALT and AST levels in the serum sample, ALT Activity Colorimetric assay kit (K752-100, Biovision, China) and AST Activity Colorimetric assay kit (K753-100, Biovision) were used respectively.

**Supplementary References**

1. Castro MM, Tanus-Santos JE, Gerlach RF. Matrix metalloproteinases: targets for doxycycline to prevent the vascular alterations of hypertension. *Pharmacol Res* **64**, 567-572 (2011).

2. Catapano MC, Tvrdy V, Karlickova J, Mercolini L, Mladenka P. A simple, cheap but reliable method for evaluation of zinc chelating properties. *Bioorg Chem* **77**, 287-292 (2018).

3. Kim Y*, et al.* Small molecule-mediated reprogramming of human hepatocytes into bipotent progenitor cells. *J Hepatol* **70**, 97-107 (2019).
