# Supplementary material for: Bioengineered liver crosslinked with nano-graphene oxide enables efficient liver regeneration via MMP suppression and immunomodulation
Source: Nat Commun. 2023 Feb 13;14:801. doi: 10.1038/s41467-023-35941-2 (PMC9925774; doi:10.1038/s41467-023-35941-2)
Supplement: Supplementary file 2 — Description of additional Supplementary File [file 41467_2023_35941_MOESM2_ESM.pdf]

<u>Descriptions of additional supplementary files</u>

Supplementary Data 1: LC/MS analysis of native liver and dECM liver scaffold
