# Supplementary material for: Bioengineered liver crosslinked with nano-graphene oxide enables efficient liver regeneration via MMP suppression and immunomodulation
Source: Nat Commun. 2023 Feb 13;14:801. doi: 10.1038/s41467-023-35941-2 (PMC9925774; doi:10.1038/s41467-023-35941-2)
Supplement: Supplementary file 4 — Reporting Summary [file 41467_2023_35941_MOESM4_ESM.pdf]

nature portfolio

Corresponding author(s): Kyung-Sun Kang

Last updated by author(s): Dec 15, 2022

Reporting Summary

Nature Portfolio wishes to improve the reproducibility of the work that we publish. This form provides structure for consistency and transparency in reporting. For further information on Nature Portfolio policies, see our Editorial Policies and the Editorial Policy Checklist.

Statistics

For all statistical analyses, confirm that the following items are present in the figure legend, table legend, main text, or Methods section.

n/a	Confirmed	
☐	☒	The exact sample size (n) for each experimental group/condition, given as a discrete number and unit of measurement
☐	☒	A statement on whether measurements were taken from distinct samples or whether the same sample was measured repeatedly
☐	☒	The statistical test(s) used AND whether they are one- or two-sided<br>*Only common tests should be described solely by name; describe more complex techniques in the Methods section.*
☒	☐	A description of all covariates tested
☐	☒	A description of any assumptions or corrections, such as tests of normality and adjustment for multiple comparisons
☐	☒	A full description of the statistical parameters including central tendency (e.g. means) or other basic estimates (e.g. regression coefficient) AND variation (e.g. standard deviation) or associated estimates of uncertainty (e.g. confidence intervals)
☐	☒	For null hypothesis testing, the test statistic (e.g. F, t, r) with confidence intervals, effect sizes, degrees of freedom and P value noted<br>*Give P values as exact values whenever suitable.*
☒	☐	For Bayesian analysis, information on the choice of priors and Markov chain Monte Carlo settings
☒	☐	For hierarchical and complex designs, identification of the appropriate level for tests and full reporting of outcomes
☒	☐	Estimates of effect sizes (e.g. Cohen's d, Pearson's r), indicating how they were calculated

Our web collection on statistics for biologists contains articles on many of the points above.

Software and code

Policy information about availability of computer code

Data collection	Image collection: NIS-Elements AR (version 3.2) and EZ-C1 software (version 3.80) (Nikon, Japan)<br>Rheological analysis: UniVert (version) (CellScale, Canada)<br>qPCR analysis: Applied Biosystems 7500 (version 2.3)<br>Microplate reader: i-control (version 1.9)<br>Flow cytometry: BD CellQuest Pro (version 6.0)
Data analysis	Graph design and statistical analysis: Prism 9 (version 9.3.0), Origin Pro (version 9.8.0.200), Microsoft Excel (version 16.0)<br>Immunofluoresence: ImageJ (version 1.53)<br>Proteomics: MaxQuant (version 1.6.10.54), STRING (version 11.0)<br>Flow cytometry: FlowJo (version 10.6.1)

For manuscripts utilizing custom algorithms or software that are central to the research but not yet described in published literature, software must be made available to editors and reviewers. We strongly encourage code deposition in a community repository (e.g. GitHub). See the Nature Portfolio guidelines for submitting code & software for further information.

Data

Policy information about availability of data

All manuscripts must include a data availability statement. This statement should provide the following information, where applicable:

- Accession codes, unique identifiers, or web links for publicly available datasets
- A description of any restrictions on data availability
- For clinical datasets or third party data, please ensure that the statement adheres to our policy

There are no data relevant to accession codes or unique identifiers that are not publicly available. All generated data in this paper are provided as Source data. For mass spectrometry analysis, rattus norvegicus database was downloaded from Uniprot (released on 2017/12/20: 31,571 sequences and 17,286,506 residues).

Human research participants

Policy information about studies involving human research participants and Sex and Gender in Research.

Reporting on sex and gender	N/A
Population characteristics	N/A
Recruitment	*Describe how participants were recruited. Outline any potential self-selection bias or other biases that may be present and how these are likely to impact results.*
Ethics oversight	N/A

Note that full information on the approval of the study protocol must also be provided in the manuscript.

Field-specific reporting

Please select the one below that is the best fit for your research. If you are not sure, read the appropriate sections before making your selection.

☒ Life sciences ☐ Behavioural & social sciences ☐ Ecological, evolutionary & environmental sciences

For a reference copy of the document with all sections, see nature.com/documents/nr-reporting-summary-flat.pdf

Life sciences study design

All studies must disclose on these points even when the disclosure is negative.

Sample size	Sample sizes for each experiment were chosen according to our previous study (Molecular Therapy, 2020, 20(2):466-478) and the standards of the field, and not predetermined based on statistical methods. In this study, the results were obtained from 4 biological replicates for in vitro or ex vivo experiments and 5 biological replicates for in vivo experiments.
Data exclusions	No data were excluded.
Replication	The biological replicates were stated in each figure legend (n=). Three technical replications were performed throughout the study. To ensure the data reproducibility, all experiments were repeated three times independently.
Randomization	Throughout the study, the samples were randomly allocated to minimize the effects of subjective bias. In animal experiments, all mice were maintained under standard environmental condition and randomly assigned for each group. For transplantation, the dECM scaffolds and MBLs were divided into the same size and randomly assigned for each group.
Blinding	Investigators were blinded to group allocation and analysis of in vitro, ex vivo and in vivo experiments.

Reporting for specific materials, systems and methods

We require information from authors about some types of materials, experimental systems and methods used in many studies. Here, indicate whether each material, system or method listed is relevant to your study. If you are not sure if a list item applies to your research, read the appropriate section before selecting a response.

Materials & experimental systems

n/a	Involved in the study
	☒ Antibodies
	☒ Eukaryotic cell lines
☒	Palaeontology and archaeology
	☒ Animals and other organisms
☒	Clinical data
☒	Dual use research of concern

Methods

n/a	Involved in the study
☒	ChIP-seq
	☒ Flow cytometry
☒	MRI-based neuroimaging

Antibodies

Antibodies used

Following primary antibodies were used for immunofluorescence staining: anti-Collagen type I (GTX26308, GeneTex, USA, 1:100), anti-Collagen type IV (ab19808, Abcam, 1:100), anti-neutrophil (ab2557, Abcam, 1:100), anti-F4/80 (ab6640, Abcam, 1:100), anti-CCR7 (ab221209, Abcam, 1:100), anti-iNOS (ab15323, Abcam, 1:100), anti-CD206 (ab8918, ab64693, Abcam, 1:100), anti-CD163 (ab182422, Abcam, 1:100), anti-MMP-1 (MAB901, R&D systems, USA, 1:100), anti-MMP-2 (AB19015, Sigma-Aldrich, 1:100), anti-MMP-9 (AB19016, Sigma-Aldrich, 1:100), anti-TIMP-1 (sc-21734, Santa Cruz Biotechnology, USA, 1:50), anti-TIMP-2 (sc-5539, Santa Cruz Biotechnology, 1:50), anti-α-SMA (ab184675, Abcam, 1:100), anti-CD86 (ab239075, Abcam, 1:100), anti-CD68 (ab955, Abcam, 1:100), anti-CD4 (130-120-819, Miltenyi Biotec, Germany, 1:50), anti-FOXP3 (130-120-674, Miltenyi Biotec, 1:50), anti-ALB (GTX102419, Genetex, USA, 1:100), anti-CK18 (MAB3234, Merck Millipore, USA, 1:100), anti-ZO-1 (40-2200, Invitrogen, USA, 1:100), anti-HNF4a (MA1-199, Invitrogen, 1:100), anti-CD31 (ab28364, Abcam, 1:100), anti-cleaved Caspase3 (9664, Cell signaling technology, USA, 1:100), anti-Desmin (sc-23879, Santa Cruz Biotechnology, 1:50), and anti-Cytoglobin (GTX117571, GeneTex, 1:100)
Following antibodies were used for flow cytometry: anti-CD14 (555397), anti-CD86 (555659), anti-CD163 (563887) and anti-CD206 (555954), which were all purchased from BD bioscience.

Fluorescent-dye conjugated secondary antibodies were used as follows: Alexa Flour 488-labeled (A11001, A11006, A11008), 594-labeled (A11005, A11012), 647-labeled (A21235) and 488-labeled streptavidin (S11223) (all purchased from Invitrogen).

Validation

All antibodies listed above are commercially available and have been verified by their manufacturer. Information regarding antibody validation can be found on the manufacturer's website as provided below.

1. Antibodies from Abcam
- anti-Collagen type IV (ab19808). Rabbit polyclonal; suitable for IHC; reacts with mouse and predicted to work with rat; https://www.abcam.com/collagen-iv-antibody-ab19808.html
- anti-CCR7 (ab221209). Rabbit monoclonal (Y59); suitable for WB, IP, ICC/IF and Mass Cytometry; reacts with human and predicted to work with mouse, rat and monkey; https://www.abcam.com/ccr7-antibody-y59-low-endotoxin-azide-free-ab221209.html
- anti-iNOS (ab15323). Rabbit polyclonal (RM1017); suitable for IHC and WB; reacts with mouse and recombinant fragment; https://www.abcam.com/inos-antibody-ab15323.html
- anti-CD206 (ab8918). Mouse monoclonal (15-2); suitable for IHC; reacts with human; https://www.abcam.com/mannose-receptor-antibody-15-2-ab8918.html
- anti-CD206 (ab64693). Rabbit polyclonal (EPR22489-7); suitable for IHC, WB and ICC; reacts with mouse, rat and human; https://www.abcam.com/mannose-receptor-antibody-ab64693.html
- anti-CD163 (ab182422). Rabbit monoclonal (EPR19518); suitable for Flow cytometry, IHC and WB; reacts with mouse, rat and human; https://www.abcam.com/cd163-antibody-epr19518-ab182422.html
- anti-α-SMA (ab184675). Alexa Flour 488 mouse monoclonal (1A4); suitable for Flow cytometry and ICC/IF; reacts with rat and predicted to work with mouse and human; https://www.abcam.com/alexa-fluor-488-alpha-smooth-muscle-actin-antibody-1a4-ab184675.html
- anti-CD86 (ab239075). Rabbit monoclonal (EPR21962); suitable for WB, IP, ICC/IF and flow cytometry; reacts with human; https://www.abcam.com/cd86-antibody-epr21962-ab239075.html
- anti-CD68 (ab955). Mouse monoclonal (KP1); suitable for WB, IHC and ICC/IF; reacts with human; https://www.abcam.com/cd68-antibody-kp1-ab955.html
- anti-CD31 (ab28364). Rabbit polyclonal (EPR21962); suitable for IHC; reacts with human and predicted to work with mouse and pig; https://www.abcam.com/cd31-antibody-ab28364.html

2. Antibody from R&D systems
- anti-MMP-1 (MAB901). Mouse monoclonal (36665); suitable for IHC, IP and WB; reacts with human; https://www.rndsystems.com/products/human-mmp-1-antibody-36665_mab901

3. Antibodies from Sigma-Aldrich
- anti-MMP-2, catalytic domain (AB19015). Rabbit polyclonal; suitable for IP and WB; This antibody has also been used for IHC (European Journal of Obstetrics & Gynecology and Reproductive Biology, 2015, 184:7-12; Journal of Molecular and Cellular Cardiology, 2016, 94:153-161; Reproductive Sciences, 2021, 28:2650-2660); reacts with human, mouse and rat; https://www.merckmillipore.com/KR/ko/product/Anti-MMP-2-Antibody-catalytic-domain,MM_NF-AB19015?ReferrerURL=https%3A%2F%2Fwww.google.com%2F&bd=1
- anti-MMP-9, catalytic domain (AB19016). Rabbit polyclonal; suitable for IHC, IP and WB; reacts with human, mouse and rat; https://www.merckmillipore.com/KR/ko/product/Anti-MMP-9-Antibody-Catalytic-domain,MM_NF-AB19016

4. Antibodies from Santa Cruz Biotechnology
- anti-TIMP-1 (sc-21734). Mouse monoclonal (2A5); suitable for IHC, IF and WB; reacts with human, mouse and rat; https://

www.scbt.com/ko/p/timp-1-antibody-2a5
- anti-TIMP-2 (sc-5539). Rabbit polyclonal (H-140); suitable for IHC and WB; reacts with human, mouse and rat; https://www.scbt.com/p/timp-2-antibody-h-140?requestFrom=search
- anti-Desmin (sc-23879). Mouse monoclonal (RD301); suitable for WB, IP, IF and IHC; reacts with human, mouse and rat; https://www.scbt.com/ko/p/desmin-antibody-rd301

5. Antibody from GeneTex
- anti-ALB (GTX102419). Mouse monoclonal; suitable for WB, ICC/IF and IHC; reacts with human, mouse and rat; https://www.genetex.com/Product/Detail/Albumin-antibody/GTX102419
- anti-Collagen I (GTX26308). Mouse monoclonal; suitable for WB, ICC/IF and IHC; reacts with human, mouse and rat; https://www.genetex.com/Product/Detail/Collagen-I-antibody-COL-1/GTX26308
- anti-Cytoglobin (GTX117571). Rabbit polyclonal; suitable for WB, ICC and IF; eacts with human, mouse and rat; https://www.genetex.com/Product/Detail/Cytoglobin-antibody/GTX117571

6. Antibody from Merck Millipore
- anti-CK18 (MAB3234). Rabbit polyclonal (RGE53); suitable for WB, ICC/IF, Flow cytometry and IHC; reacts with human, mouse and rat; https://www.merckmillipore.com/KR/ko/product/Anti-Cytokeratin-18-Antibody-clone-RGE53,MM_NF-MAB3234?ReferrerURL=https%3A%2F%2Fwww.google.com%2F&bd=1

7. Antibodies from Invitrogen
- anti-ZO-1 (40-2200). Rabbit polyclonal; suitable for WB, ICC/IF and IHC; reacts with human, mouse, dog and rat; https://www.thermofisher.com/antibody/product/ZO-1-Antibody-Polyclonal/40-2200
- anti-HNF4a (MA1-199). Mouse Monoclonal (K9218); suitable for IP, ELISA, WB, ICC/IF, Flow cytometry and IHC; reacts with human, mouse and rat; https://www.thermofisher.com/antibody/product/HNF4A-Antibody-clone-K9218-Monoclonal/MA1-199

8. Antibody from Cell signaling technology
- anti-cleaved Caspase3 (9664). Rabbit polyclonal (5A1E); suitable for WB, IP, ICC/IF, Flow cytometry and IHC; reacts with human, mouse, monkey and rat; https://www.cellsignal.com/products/primary-antibodies/cleaved-caspase-3-asp175-5a1e-rabbit-mab/9664

9. Antibodies from Miltenyi Biotec
- FITC rat anti-mouse CD4 (130-120-819). Clone GK1.5; suitable for Immunofluorescence and flow cytometry; https://www.miltenyibiotec.com/KR-en/products/cd4-antibody-anti-mouse-gk1-5.html?countryRedirected=1#fitc:30-ug-in-200-ul
- PE anti-mouse FOXP3 (130-120-674). Clone REA568; suitable for Immunofluorescence and flow cytometry; https://www.miltenyibiotec.com/KR-en/products/treg-detection-kit-cd4-cd25-foxp3-mouse.html?countryRedirected=1#gref

10. Antibodies from BD bioscience
- FITC mouse anti-human CD14 (555397). Clone M5E2; https://www.bdbiosciences.com/en-nz/products/reagents/flow-cytometry-reagents/research-reagents/single-color-antibodies-ruo/fitc-mouse-anti-human-cd14.555397
- PE-Cy5 mouse anti-human CD86 (555659). Clone 2331; https://www.bdbiosciences.com/en-nz/products/reagents/flow-cytometry-reagents/research-reagents/single-color-antibodies-ruo/pe-cy-5-mouse-anti-human-cd86.555659
- PerCP-Cy5.5 mouse anti-human CD163 (563887). Clone GHI/61; https://www.bdbiosciences.com/en-nz/products/reagents/flow-cytometry-reagents/research-reagents/single-color-antibodies-ruo/percp-cy-5-5-mouse-anti-human-cd163.563887
- PE mouse anti-human CD206 (555954). Clone 19.2; https://www.bdbiosciences.com/en-nz/products/reagents/flow-cytometry-reagents/research-reagents/single-color-antibodies-ruo/pe-mouse-anti-human-cd206.555954

Eukaryotic cell lines

Policy information about cell lines and Sex and Gender in Research

Cell line source(s)	Human PBMCs were purchased from Lonza (CC-2702). Human primary ECs were sourced from ATCC (CRL-1730). Human CdH and miHep cells were originally derived in the lab of Pf. Dongho Choi. BALB/c mouse primary liver sinusoidal endothelial cells were obtained from Cell Biologics (BALB-5017).
Authentication	All cell lines were either purchased directly from respective manufacturer or directly mailed from Pf. Choi's lab, but we did not independently authenticate them.
Mycoplasma contamination	All cells were negative for mycoplasma infection. Mycoplasma contamination was rountely tested with e-Myco™ VALiD Mycoplasma PCR Detection Kit (iNtRON, Republic of Korea).
Commonly misidentified lines (See ICLAC register)	No commonly misidentified cell lines were used in the study.

Animals and other research organisms

Policy information about studies involving animals; ARRIVE guidelines recommended for reporting animal research, and Sex and Gender in Research

Laboratory animals	6 week-old Sprague Dawley rats (200-250g) were used for fabrication of dECM liver scaffolds. 6 week-old or 8 week-old male BALB/c mice were used for transplantation of dECM scaffolds and MBLs. Please see Methods section in article for further details. All animals were purchased from DBL (Republic of Korea) and maintained on 12 hours of light/dark cycles at 18-23°C, 45% humidity.

Wild animals	No wild animals were used in this study.
Reporting on sex	To establish acute or chronic liver failure mouse models, male mice were used to preclude the effects of sex hormone since liver metabolism and inflammatory responses are highly influenced by estrogen levels and activity of estrogen receptor (Hepatobiliary & Pancreatic Diseases International, 2020, 19(5):429-434; Endocrine Reviews, 2007, 28(5):521-574).
Field-collected samples	No field-collected samples were used in this study.
Ethics oversight	All in vivo experiments were conducted with the approval of Seoul National University Institutional Animal Care and Use Committee: Fabrication of decellularized rat liver scaffolds and transplantation of crosslinked scaffolds (SNU-191208-1) and transplantation of mouse bioengineered liver produced with crosslinked scaffolds (SNU-211130-2). The experiments were designed in compliance with the ARRIVE guidelines.

Note that full information on the approval of the study protocol must also be provided in the manuscript.

Flow Cytometry

Plots

Confirm that:

- [ ] The axis labels state the marker and fluorochrome used (e.g. CD4-FITC).
- [ ] The axis scales are clearly visible. Include numbers along axes only for bottom left plot of group (a 'group' is an analysis of identical markers).
- [ ] All plots are contour plots with outliers or pseudocolor plots.
- [x] A numerical value for number of cells or percentage (with statistics) is provided.

Methodology

Sample preparation	CD14+ cells sorted from human PBMCs were seeded into each scaffold and polarized into either M1 or M2 like macrophages. Appropriate negative controls were also used. Harvested cells were probed with antibodies for 15 minutes on ice. The cells were washed with PBS and subjected to flow cytometry analysis. Please see Methods section in article for further details.<br>CBA assay was also performed with flow cytometry. Following the manufacturer's instructions, the serum samples collected from mice transplanted with each scaffolds or MBLs were incubated with capture bead mixtures PE detection reagents for 2 hours at room temperature. After washed with wash buffer, the samples were subjected to flow cytometry analysis.
Instrument	Flow cytometry data were collected using BD FACS Calibur (BD bioscience).
Software	BD CellQuest Pro (version 6.0) was used for data collection during flow cytometry analysis and the results were analyzed by using FlowJo (version 10.6.1).
Cell population abundance	CD14+ populations were obtained by using magnetic activated cell sorting(MACS). No fluorescence activated cell sorting (FACS) was performed in this study.
Gating strategy	CD14+ cells polarized into M1 or M2 like macrophages were subjected to flow cytometry. After FSC-A/SSC-A live cell gating, CD14-FITC was gated for distinguishing pan-macrophage populations. The boundary for negative and positive populations was determined by negative control populations. Among CD14+ populations, M1 populations were identified based on the expression of CD86 and CD163+ CD206+ (double positive) populations were regarded as M2 populations.<br>During CBA assay, acquisition strategy was established with cytometer setup beads according to the manufacturer's protocol (https://www.bdbiosciences.com/en-nz/products/reagents/immunoassay-reagents/cba/cba-kits/mouse-inflammation-kit.552364). Based on the template, the singlet bead populations were first gated using FSC-H/SSC-H dot plot, followed by gating each capture bead in FL2-H/ FL3-H dot plot (each capture bead can be distinguished by fluorescence intensity). After gating, corresponding MFI of FL2-H was quantified. By using MFI values, the concentration of each protein in the serum samples was calculated according to the standard curve.

- [x] Tick this box to confirm that a figure exemplifying the gating strategy is provided in the Supplementary Information.
